# High resolution multi-locus sequence typing scheme for *Giardia duodenalis* assemblage B outbreak and population analysis

**DOI:** 10.1371/journal.pntd.0014528

**Published:** 2026-07-15

**Authors:** Christian Klotz, Katja Winter, Marc W. Schmid, Stephan Fuchs, Anna Rosa Sannella, Umer Chaudhry, Jacinto Gomes, Ralf Ignatius, Toni Aebischer, Martha Betson, Karin Troell, Simone M. Cacciò

**Affiliations:** 1 Department of Infectious Diseases, Unit for Mycotic and Parasitic Agents and Mycobacteria, Robert Koch-Institute, Berlin, Germany; 2 Bioinformatics Core Facility (MF1), Robert Koch-Institute, Berlin, Germany; 3 MWSchmid GmbH, Glarus, Switzerland; 4 Department of Infectious Diseases, Istituto Superiore di Sanità, Rome, Italy; 5 Discipline of Comparative Biomedical Sciences, School of Veterinary Medicine, University of Surrey, Surrey, United Kingdom; 6 Elvas School of Biosciences, Polytechnic Institute of Portalegre, Portalegre, Portugal; 7 VALORIZA – Research Centre for Endogenous Resources Valorization, Portalegre, Portugal; 8 MVZ Labor 28, Berlin, Germany; 9 Institute of Microbiology, Infectious Diseases and Immunology, Charité-University Medicine Berlin, Campus Benjamin Franklin, Berlin, Germany; 10 Swedish Veterinary Agency, Uppsala, Sweden; 11 Norwegian Veterinary Institute, Ås, Norway; Georgetown University, UNITED STATES OF AMERICA

## Abstract

*Giardia duodenalis* represents a species complex of tetraploid protozoan parasites that infect the small intestine of mammals. Human giardiasis is a widespread gastrointestinal disease predominantly caused by two of eight genetically distinct *G. duodenalis* groups, termed assemblages A and B. These two assemblages differ in their host preferences, grade of genome identity as well as frequency of allelic sequence heterogeneity (ASH), therefore assemblage-specific typing schemes are needed for epidemiological purposes such as outbreak investigations and source attribution. Here, we used whole genome datasets of assemblage B parasites derived from 18 axenically cultured patient isolates to identify genomic markers for molecular typing. Of the 42 identified genomic loci, 20 were selected to design primer sets for a nested PCR and sequencing approach, and a final set of seven markers was included in a new multi locus sequence typing (MLST) scheme. The MLST scheme was successfully applied to 109 (75%) out of 146 tested assemblage B samples. As assemblage B is characterized by high ASH, the analysis included calling of ASH positions as part of the genotyping approach to distinguish isolates. In epidemiologically unrelated samples (n = 80), the MLST scheme revealed a bipartite population structure comprised of organisms with low or high ASH. On the other hand, samples from a waterborne outbreak (n = 16) and samples from six out of eight separate epidemiologically linked cases formed separate clusters that were distinct from unrelated sporadic cases. These results indicate that the new typing scheme is informative and could assist future epidemiological studies of *G. duodenalis* assemblage B.

## Author summary

Giardiasis is a gastrointestinal disease of public health concern worldwide. The causative agent, *Giardia duodenalis*, represents a species complex of eight genetically very distinct groups, assigned assemblages. Humans are predominantly infected by assemblages A and B, which both may have zoonotic potential. To classify isolates for epidemiological purposes, such as outbreak investigations, adequate typing schemes are necessary. For assemblage B, the development of such typing schemes has been hampered by the lack of genome datasets and high levels of allelic sequence heterogeneity (ASH) in its tetraploid genome. Here, based on comparative analysis of 18 assemblage B genomes, a new typing scheme based on seven genomic markers was developed. Successful application of this scheme to 109 isolates revealed a complex bipartite population structure with some isolates showing high ASH and, unexpectedly, others containing no or low ASH. In addition, samples from epidemiologically linked cases clustered together and were distinct from sporadic cases. In conclusion, this work presents new insights into the complex population structure of *G. duodenalis* assemblage B and provides a typing scheme with improved resolution for epidemiological investigations. It also provides a starting point to develop more sophisticated and automated typing strategies as genomic information for *G. duodenalis* is growing.

## Introduction

Giardiasis is a widespread gastrointestinal disease of public health relevance, caused by the protozoan parasite *Giardia duodenalis* (syn. *G. intestinalis*, *G. lamblia*). The parasite represents a species complex comprised of eight genetically distinguishable groups, termed assemblage A to H [1,2]. Of these, assemblage A and B are considered to have zoonotic potential as they are found in a variety of mammals and represent the dominant assemblage types found in humans. The real risk of zoonotic transmission is still debated, partly because current typing schemes lack the appropriate resolution for source attribution and other epidemiological purposes [1–4]. The remaining assemblages show more narrow host species distributions, with assemblages C and D found in canids, E in hoofed animals, F in felids, G in rodents, and H in pinnipeds. Assemblage E has been occasionally detected in humans, but at much lower frequency than assemblages A and B [1,4]. Further genetic diversity exists within assemblages, with recognition of sub-assemblages and genotypes. The taxonomy of *G. duodenalis* is still debated [1,4,5], and some experts have suggested to rename the assemblages with appropriate species names, e. g., assemblage AI (= *G. duodenalis duodenalis*), AII (= *G. duodenalis intestinalis*), assemblage AIII (*G. duodenalis cervus)*, assemblage B (= *G. enterica*) [3,4]. In the context of this work, however, we will refer to the assemblage nomenclature as it is still the most widely used.

Assemblages A and B are genetically very diverse, sharing only approximately 70% nucleic and amino acid similarity at the genome level [5,6]. *Giardia* is characterized by the presence of two diploid nuclei (i.e., is functionally a tetraploid organism) in its vegetative form (the trophozoite). Nuclei can accumulate mutations independently of each other, which can generate sequence heterogeneity among the four copies of each gene present in a single cell. This is known as allelic sequence heterogeneity (ASH), and the existing data suggest ASH to be present at much lower frequency in assemblage A than in assemblage B [1,4,7].

Previous typing schemes have been mostly based on genetic information derived from PCR and sequencing of housekeeping or *Giardia*-specific genes. Typical marker genes include triosephosphate isomerase (*tpi*), glutamate dehydrogenase (*gdh*), and beta-giardin (*bg*) [2,8–11]. These markers have been used to further subdivide assemblages into several sub-groups, namely sub-assemblages AI, AII and AIII for assemblage A, and sub-assemblages BIII and BIV for assemblage B [1,4]. However, the existence of sub-assemblages BIII and BIV within assemblage B in human samples, originally suggested from allozyme electrophoresis analyses [12], is debated as analysis of the DNA marker sequences gave conflicting results [8,13]. Due to their limited resolving power, analyses based on these genes alone are not fully appropriate to distinguish isolates for epidemiological purposes or source attribution. In addition, drastic differences in ASH content indicate that separate typing schemes are needed to distinguish individual isolates within assemblage A and B.

For assemblage A, a new typing scheme based on six genomic loci has been introduced [14] and robustly tested for its suitability to distinguish outbreak isolates from sporadic, epidemiologically unrelated infections [7,15]. For assemblage B, more complex typing schemes have been suggested based on additional markers [16,17]. Practical applicability, however, has not yet been confirmed in interlaboratory comparisons or else showed inconsistent results [18].

In the present study, we mined whole genomes of 18 axenically culturable *G. duodenalis* assemblage B isolates to identify genomic regions suitable for developing a new multi-locus typing scheme (MLST) with acceptable resolution for epidemiological purposes, such as outbreak investigations. A final set of seven markers was selected and their resolution and suitability to identify epidemiologically linked samples was tested on a large collection of assemblage B isolates.

## Materials and methods

### Ethics statement

No patient data was collected and used in the present study. Under the Protection against Infection Act, Giardiasis is a notifiable disease in Germany and the study conducted in accordance with the Protection against Infection Act’s §13. In this context, informed consent was not required.

#### Parasite isolates*.*

Whole genome sequence data derived from DNA of 16 axenic assemblage B isolates of an in-house biobank [5,19] and of two isolates, GS (GCA_011634595.1) and BAH15c1 (GCA_001543975.1), from public resources [6,20–22] were used.

Additionally, 141 DNAs extracted from fecal samples were included (see Table A in S1 Appendix for more information on the samples). This comprised 96 samples from sporadic human giardiasis cases and 22 samples from chronically infected patients who provided more than one stool sample (longitudinal cases) collected at RKI, along with four samples from sporadic human giardiasis cases and three samples of animal origin (one monkey, one cat, one chinchilla), which were provided by the Swedish Veterinary Agency (SVA), Sweden, the University of Surrey (UoS), UK and the National Institute for Agricultural and Veterinary Research (INIAV), Portugal. Finally, 16 samples from a previously described assemblage B waterborne outbreak in Italy [23] were also included.

#### Pairwise genetic distance of assemblage B genomes.

The genome data included five of our recent de novo assemblies derived from both long and short read sequencing [5], two de novo assemblies from public databases [21,22] and raw Illumina datasets from an additional 11 isolates [19]. The genome of isolate P424 was chosen as reference. Raw reads were trimmed with fastp (version 0.20.0, [24]) and aligned to the reference genome using bowtie2. Only alignments with a minimal mapping quality of 10 were kept (version 2.4.5, [25]). Alignment statistics were extracted from the console output (logs). Single nucleotide polymorphisms (SNPs) were identified with bcftools (version 1.18, [26]) and filtered for a minimal quality of 20. SNPs were then filtered for a coverage between 30 and 1000 in all samples. Pairwise genetic distances were calculated as the fraction of alleles that differed between two individuals, ignoring SNPs for which no data were available for one or both individuals. Distance data were visualized as dendrogram using as.dendrogram in R. The BAH15c.1 isolate was not included in the analysis since raw read data were not found in the GenBank repository.

#### Identification of suitable typing regions from whole genome sequence data.

**Fig 1:**
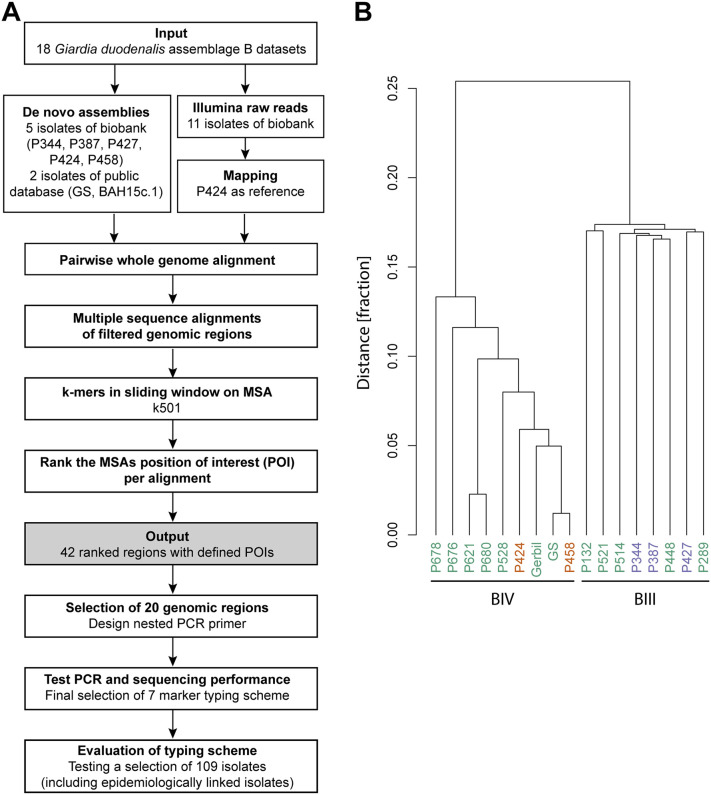
Panel A, step-by-step diagram illustrating the analytical workflow used to identify genomic regions able to distinguish assemblage B isolates; panel B, dendrogram showing the genetic relationships among the 18 assemblage B genomes used in this work.

The rationale employed to identify potential regions suitable for primer design for typing purposes is presented in Fig 1 (Panel A). The respective script is available at GitHub [27]. Briefly, each whole genome sequence (WGS) assembly input file was used for pairwise alignment against the reference genome by using the nucmer tool in MUMer (hmmer v3.3, [28]). Alignment information for all samples was combined into one matrix, filtered and sorted for length and presence of alignment positions in all samples. Filtered regions were used to generate multiple sequence alignments (MSA) using MAFFT (v7.471, [29]). A sliding window of 501 bp was used to identify regions able to distinguish all samples. We define a position of interest (POI) as the central position of a 501 bp genomic region suitable to distinguish the input sequences.

#### Molecular typing and sequence analysis.

The Primer3 algorithm implemented in the Geneious software tool (Biomatters) was used to design primers for nested PCR reactions. These primers were tested on samples previously typed as assemblage B using standard protocols for *tpi* [11], *gdh* [10] or *bg* [9] gene markers. PCR reactions were prepared in a final volume of 50 µl containing 2.5 U of DreamTaq DNA polymerase (Thermo Fisher, EP0701), 1x PCR buffer, 3 mM MgCl_2_, 200 nM dNTPs, 400 nM primer and 2 µl DNA. For the nested PCR, 1.5 µl of the first PCR was used as template. PCR conditions included 35 cycles of denaturation (30 sec at 95°C), annealing (30 sec at 58°C) and extension (60 sec at 72°C). Amplicons were analyzed on 1% agarose gel, cleaned-up with ExoSap-IT kit (Thermo Fisher #78201) and bidirectionally Sanger sequenced using the inner primers at the RKI in-house sequencing facilities. Additional samples were analyzed at SVA, UoS and ISS using the same protocols and the resulting sequences were transferred to RKI for further analysis. All sequences were manually checked, trimmed and bidirectional sequence pairs were assembled. Ambiguous positions were called after the IUPAC code with a 25% peak height cut-off, as previously described [7]. Primer sequences were excluded from the final consensus. A graphical workflow to retrieve ambiguous sequences is depicted in S1 Fig.

To determine sub-assemblages BIII and BIV, we used *tpi* reference sequences, GenBank accession number AY228628 (isolate 2924, BIII) and L02116 (isolate GS, BIV), which differ by five SNPs, and compared them to the sequences of the respective isolates. If four of the five SNP positions were identical to those in one of the references, the isolates were accordingly classified as BIII or BIV. Isolates showing ASH at these informative positions were classified as ‘undefined (ASH)’.

Multiple alignments of single or concatenated marker sequences were generated using MAFFT or Clustal Omega, and distance matrices of pairwise differences at single nucleotide positions were retrieved from the alignments. The analyses were done using software tools integrated in Geneious Prime (Biomatters) and CLC Genomics Workbench (Qiagen). The distance matrices were visualized using Prism (GraphPad software).

For further distance analysis using Ridom SeqSphere (Version 10.5.4, Ridom GmbH, Germany), the sequence data was transformed into numeric code with each IUPAC coded nucleotide represented by a specific number. A distance matrix was generated based on variant sites and results were visualized as Minimum Spanning Tree (MST) or UPGMAT-based tree.

### Data availability

All relevant data are within the manuscript and its Supporting Information files. The sequences generated in this study have been deposited in GenBank under accession numbers: PX676551 to PX677313.

## Results

### Identification of variable genomic regions in assemblage B

The 18 *G. duodenalis* assemblage B isolates from in vitro cultures were almost equally distributed between sub-assemblages BIII (8 isolates) and BIV (10 isolates), as shown in the dendrogram in Fig 1 (Panel B). Using the strategy described in Fig 1 (Panel A, see methods for details), 42 genomic regions were identified as potential marker candidates that allowed the 18 assemblage B isolates to be distinguished from one another (see S2 Appendix for msa output files). These regions were then ranked according to the number of positions of interest (POI) (see Table B in S1 Appendix).

### Pre-selection and identification of suitable markers by PCR and sequencing

**Fig 2:**
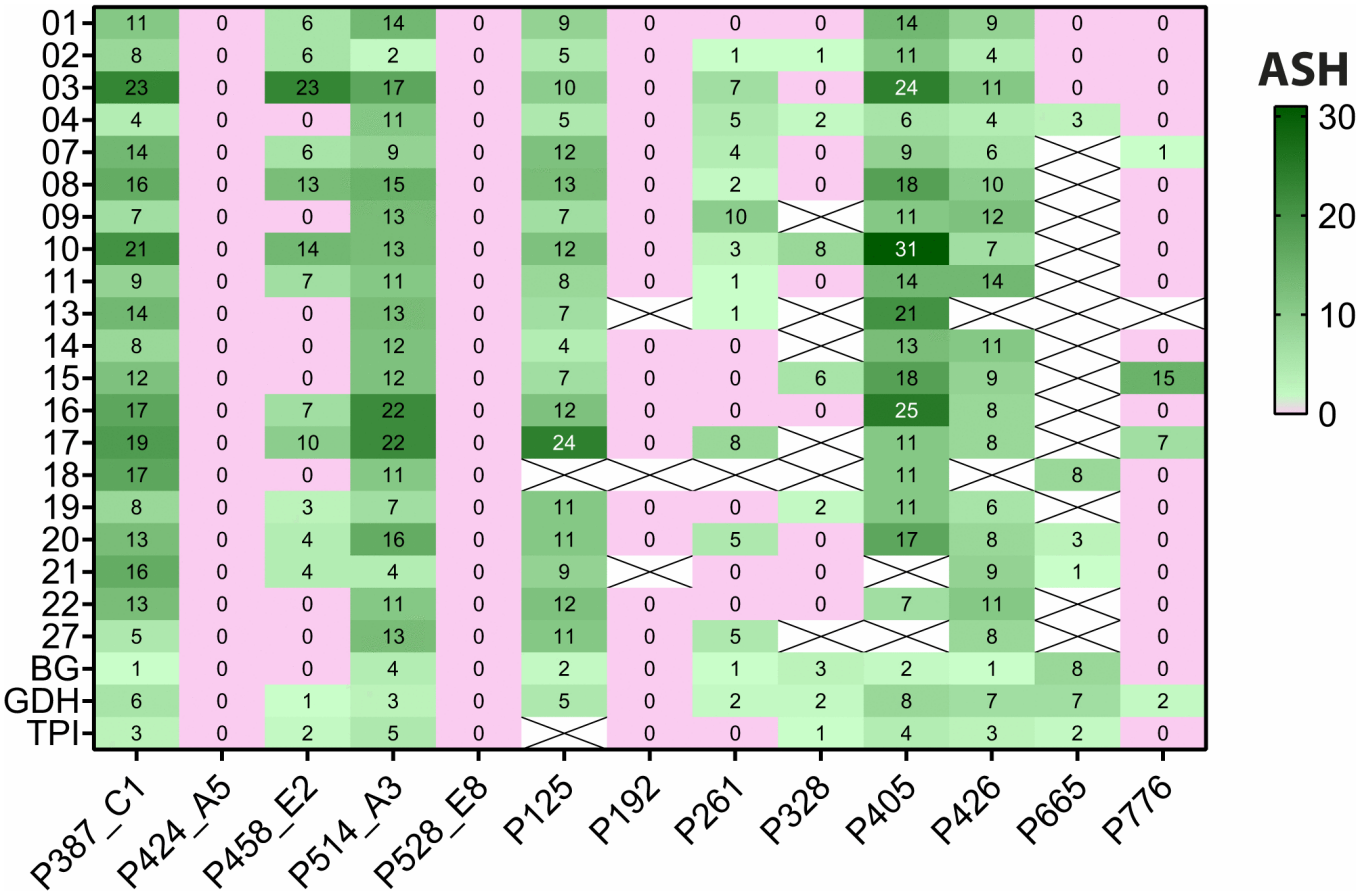
PCR-positivity and ASH characteristics for the initially selected 20 marker sequences. The isolates P387_C1, P424_A5, P458_E2, P514_A3, P528_E8 represent axenic trophozoite cultures derived by limiting dilution while the remaining samples are from fecal patient material. Failed PCRs (cross) and samples with no ASH (pink) in the respective sequences are marked.

To test applicability, we selected 20 of the 42 candidate genomic regions based on their POI rank and suitability to design primers for nested PCR. The list of the primer sequences is provided in Table C in S1 Appendix. Primers were tested on DNA extracts from five axenic cultures (positive controls) and eight stool samples, previously assigned to assemblage B based on *tpi*, *gdh* or *bg* typing. Amplicons were sequenced and compared to the reference P424 genome to confirm amplification of the correct genomic regions (S2 Fig). The overall rate of PCR positivity was high, reaching 100% for the DNA from axenic cultures and approximately 80% for the stool samples, depending on marker and sample (Fig 2). A particularly low PCR performance was observed for two samples (P328 and P665) and two markers (#13 and #18).

We next retrieved the ASH positions of the respective sequences for the 13 isolates and identified the presence of samples with homozygotic and heterozygotic sequences (Fig 2). Some samples were either completely homozygotic or completely heterozygotic over all markers, whereas other samples showed homozygotic as well as heterozygotic sequences at different markers.

**Fig 3:**
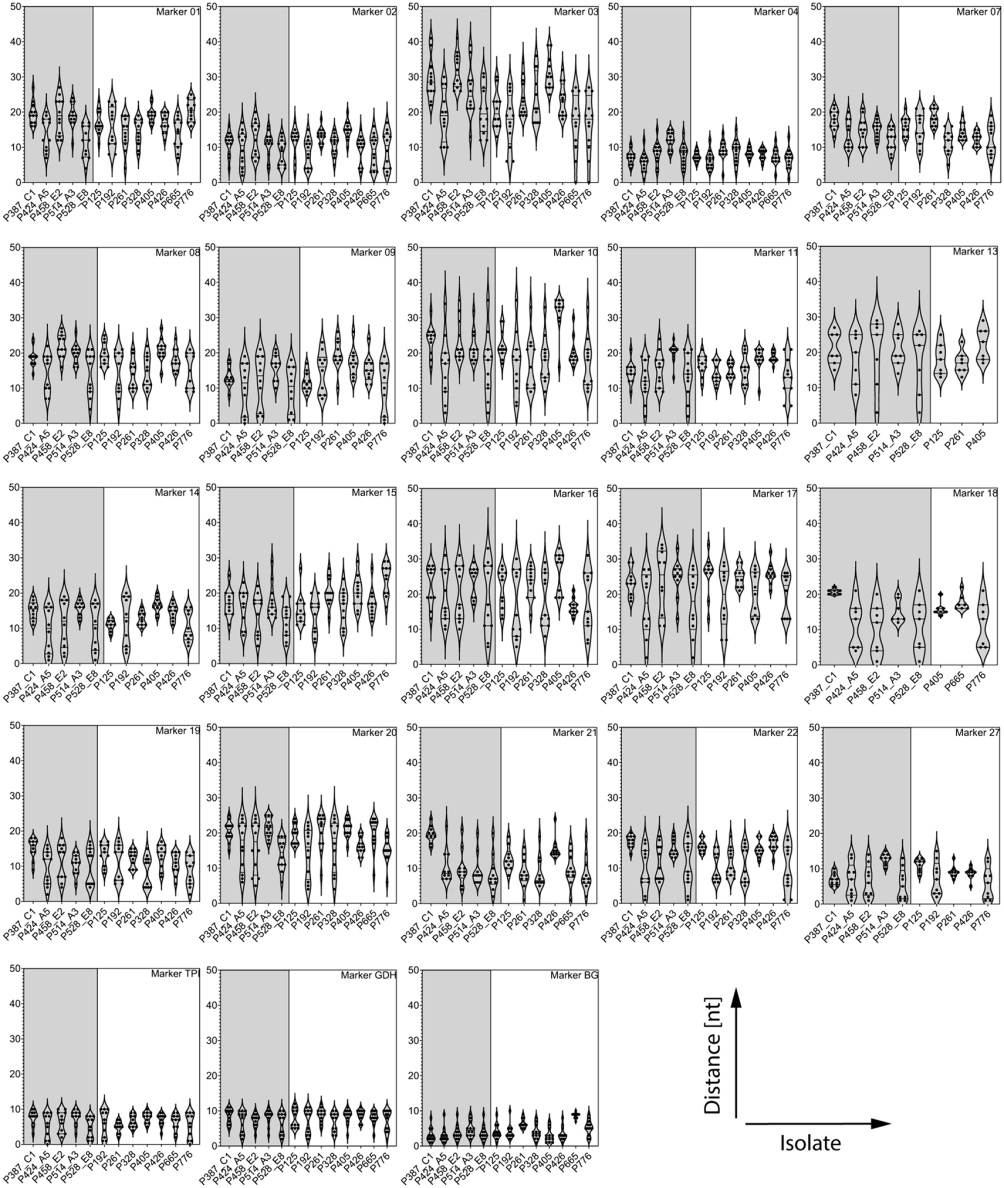
Pairwise comparison of nucleotide distance for the initially selected 20 marker sequences of 13 assemblage B isolates, including five samples from axenic isolates (in grey) and eight samples from patient fecal material. Note the overall higher distance between the 20 selected marker sequences in comparison to the standard typing gene markers *tpi*, *gdh* and *bg*.

Next, we investigated the pairwise distance between the samples, including the information on ASH positions categorized according to the IUPAC code (see methods for details). There was a much larger pairwise distance for most new markers compared to the common marker genes *tpi*, *gdh* and *bg* (Fig 3). The overall distance retrieved from genomic DNA of axenic isolates and from stool samples was similar. Notably, the distribution of pairwise distance of all markers, including the common typing marker genes, did not follow a normal distribution as indicated by violin plots (Fig 3). This indicates a non-homogenous population structure in assemblage B (see also below).

#### Assemblage B comprised homozygotic and heterozygotic organisms.

To develop an applicable MLST scheme, we selected seven markers based on PCR performance, predicted chromosomal localization and by considering whether primer sequences spanned predicted coding or non-coding regions (S2 Fig). The chromosomal localization was inferred from that predicted for the orthologous sequences in the assemblage A reference genome WB-C6 [30], as no chromosome-scale genome is yet available for assemblage B. To avoid potential linkage, markers on different chromosomes were chosen, including two markers for the larger chromosomes 4 and 5, and one marker for each of the smaller chromosomes 1, 2 and 3. The selected sequences are listed in Table 1.

**Table 1:** Selected marker for final MLST scheme.

Marker	Chromosome	Predicted function	Amplicon length
02	4	Dynein heavy chain (GD_P424_011300) and non-coding sequence*	593
07	3	Protein phosphatase (GD_P424_046620)	577
08	2	Unknown (GD_P424_019030)	570
10	5	Unknown (GD_P424_005280)	598
11	4	Methyltransferase (GD_P424_024830)	629
14	1	Ankyrin repeat protein 3 (GD_P424_049880)	536
20	5	Ankyrin repeat protein 1 (GD_P424_003020)	656

*primer for marker 02 spanning coding and 5’-prime non-coding sequence.

Overall, the MLST scheme has been applied to a total set of 146 samples, including the 13 samples used for the initial testing. For 109 samples, all seven markers could be amplified and sequenced (~75% success rate). These were used for the final analysis. The 109 samples included 68 sporadic cases, and 38 samples of 9 epidemiologically linked cases, i.e., longitudinally sampled from chronically infected patients and samples from an assemblage B waterborne outbreak in Italy. Additionally, three sporadic assemblage B samples from animals were included. The overall dataset comprised 652 variant sites over a concatenated sequence length of 3897 bp.

**Fig 4:**
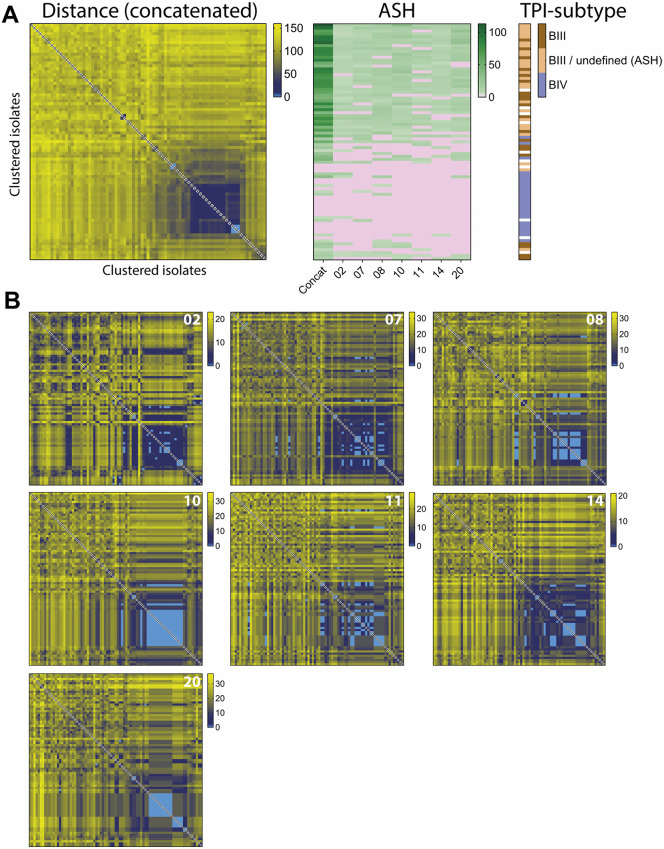
Pairwise comparison of unrelated assemblage B isolates using seven selected marker sequences. **(A)** Left panel: Matrix of pairwise distance based on the seven concatenated marker sequences, calculated using the MAFFT algorithm. Middle panel: ASH content for the concatenated sequences and for individual markers. Data are presented in the same order as the clustered isolates. Right panel: Assemblage B subtypes based on sequence analysis of the *tpi* gene marker. White bars indicate unsuccessful typing. **(B)** Distance matrix calculated for each individual marker. Isolates are ordered as in panel **A.**

To infer population structure, we first analyzed the typing results based on pairwise distance of 80 unrelated samples, including the 71 sporadic samples and one random sample of each epidemiologically linked case group, therefore excluding the remaining 29 samples with known epidemiological linkage. This revealed a nonhomogeneous distribution of pairwise distance values, with a subgroup of isolates showing more similar genotypes than others (Fig 4). This subgroup was further characterized by an absence or low number of ASH positions, and by assignment to sub-assemblage BIV by sequence analysis of the *tpi* gene marker. Importantly, a low level or an absence of ASH was not an exclusive feature of this subgroup, as no threshold value of pairwise distance was found that defined it. Instead, pairwise distance gradually increased until a homogenous mean distance value was reached with isolates possessing higher ASH values (Fig 4). Most of these samples were classified as BIII or as undefined (due to ASH) by sequence analysis of the *tpi* gene marker.

Notably, we recognized three human samples of sporadic cases within the low ASH group with identical MLST sequences (no ASH). The three animal samples were more related to the low ASH group, mostly comprised of sub-assemblage BIV samples. The cat sample showed identity with an unrelated human sample from Germany. Both samples had no ASH, and sequencing of the *tpi* gene marker of the human sample revealed a mixed BIV/BIII genotype. The monkey sample also had a low ASH content (four positions found in two of the seven markers), while the chinchilla sample had relatively high ASH content (42 positions found in four of the seven markers).

#### Suitability of the new MLST scheme to investigate linked isolates*.*

**Fig 5:**
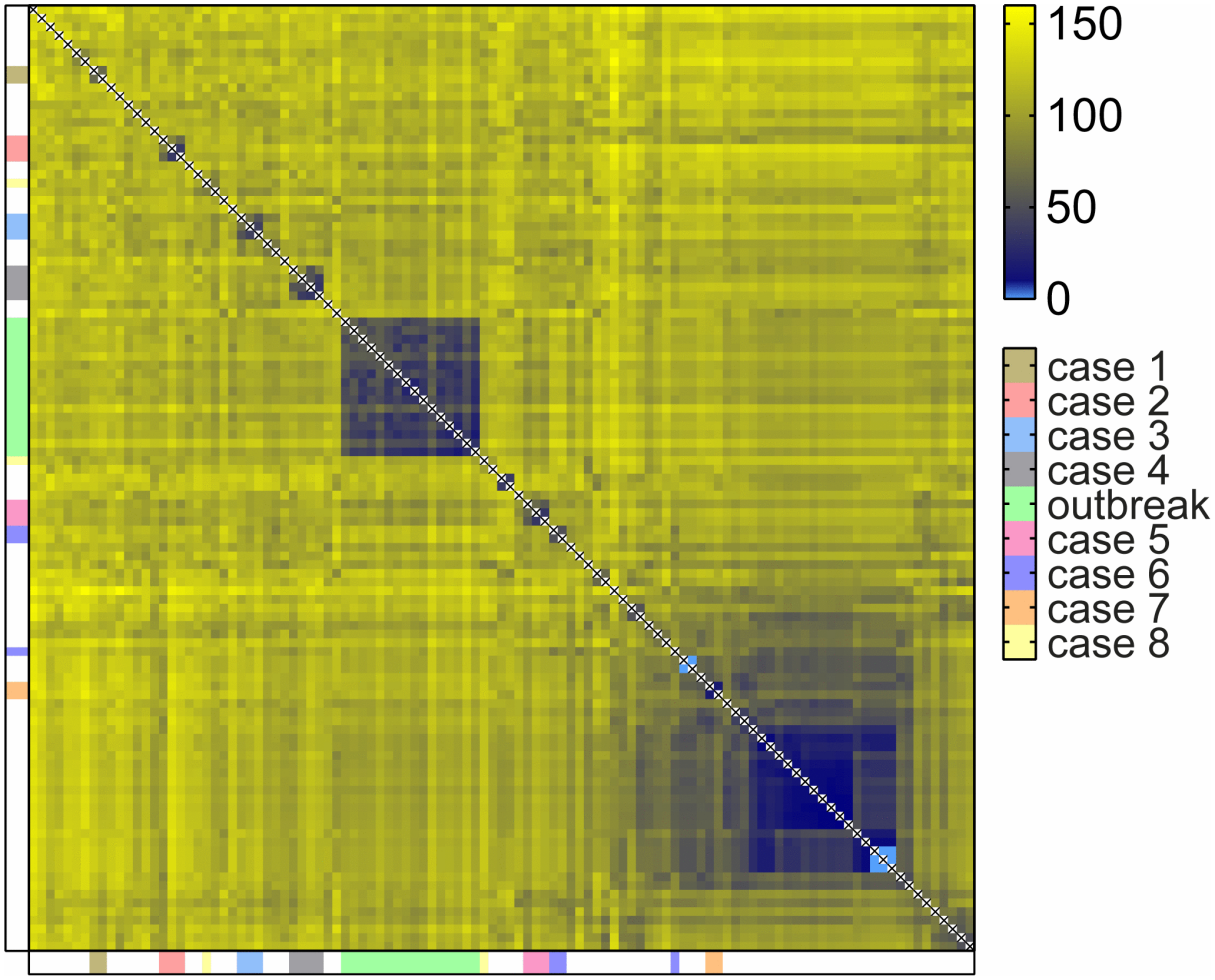
Pairwise distance comparison of unrelated and epidemiologically related assemblage B isolates using seven selected marker sequences. Alternative version of Fig 5 including sample names is provided in S5 Fig.

We next tested the potential of the new typing scheme for predicting epidemiological links by investigating samples with known relationships. The 16 samples from the Italian waterborne outbreak formed a clearly separate cluster within the high ASH group (Fig 5), which was confirmed using UPGMAT-based and MST analyses (S3 and S4 Figs). The mean distance between outbreak samples was 46 [range 17–76] while the ASH mean content was 62 [range 42–78]. Of note, 15 of the 16 samples showed a similar ASH pattern with six markers possessing significant ASH and one marker (marker 11) showing no ASH.

The clustering of related cases was confirmed by the typing results of eight cases with longitudinal samples from chronically infected patients. Samples of all related cases were clustered together (Figs 5 and S3, S4) except for one sample of case 6 and the two samples of case 8, that failed to cluster with their related samples. Intra-cluster pairwise distance is depicted in Table 2 and confirms lower mean distance of related samples compared to unrelated samples. Of note, only one of the eight cases (case 7) grouped with the isolates showing low ASH, whereas all other cases clustered with isolates showing high ASH.

**Table 2:** Mean pairwise distance of subgroups analyzed.

Subgroup/cluster	Sample size (n)	Clustered samples (n)	Mean pairwise distance [nt]	Range [nt]
All unlinked samples	80		106	0-160
High ASH^#^	40		115	37-150
Low ASH^#^	40		70	0-129
Waterborne outbreak	16	16	46	17-76
Case 1	2	2	66	n.a.
Case 2	3	3	62	36-84
Case 3	3	3	56	42-72
Case 4	4	4	58	35-89
Case 5	3	3	53	35-66
Case 6	3	2	51 [67]*	n.a. [51–89]*
Case 7	2	2	17	n.a.
Case 8	2	0	[103]*	n.a.

#ASH groups were inferred from clustered data shown in Fig 4. *Data in brackets include samples that did not cluster with their case-related samples. Not applicable (n.a.).

## Discussion

A novel typing scheme based on seven genomic marker sequences revealed a bi-partite population structure comprised of organisms with low ASH and organisms with high ASH. Importantly, samples from epidemiologically linked cases formed separate clusters that were distinct from unrelated sporadic cases, indicating that the new typing scheme could assist in future epidemiological studies of *G. duodenalis* assemblage B.

Classical genotyping markers are suitable to distinguish the different *G. duodenalis* assemblages, but do not provide the resolution necessary for epidemiological purposes, due to their limited genetic variability [4]. For assemblage A, which has an overall lower ASH content than assemblage B, a specific MLST approach using six marker genes was successfully used to identify an outbreak with anthroponotic sub-assemblage AII in a hospital [14]. This improved assemblage A typing scheme is robust and discriminates sub-assemblage AII isolates in an epidemiological context [7,14,15]. The suitability of this scheme for epidemiological investigations of zoonotic sub-assemblage AI, e.g., for source attribution, has not been tested yet. Notably, the new MLST scheme for assemblage A revealed a much higher population diversity of sub-assemblage AII versus AI [14]. This has been confirmed at the genome level and led to the hypothesis that sub-assemblages AI and AII represent two separate species [31], with evidence for recombination in sub-assemblage AII but not in AI [32].

These observations on assemblage A already highlight the complexity of the population structure of *G. duodenalis*. It is reasonable to deduce from our data that assemblage B mirrors this complexity at the genome level, which may include differences in the potential for sexual/recombinational processes. In fact, our study revealed the presence of primarily homozygous and primarily heterozygous assemblage B isolates. This agrees well with our previous results based on classical genotyping techniques [7] and with the recent discovery of an assemblage B isolate that is essentially homozygous at the genome level [5]. The homozygous isolates that showed no or low ASH were largely, but not completely, typed as sub-assemblage BIV. A previous genome-based study on a large, historical collection of isolates from Canada reported a higher diversity in assemblage B than in assemblage A; however, no analysis on ASH has been reported [33], which may be a reason for missing this aspect.

Aware of the complex issues of ASH and population structure, we pragmatically searched for an alternative procedure to enable the identification of epidemiologically related cases. The procedure included the calling of ASH positions from Sanger sequences and the generation of simple distance matrices for isolate comparison. This approach, which is agnostic of isolate relationships, revealed that isolates from a previously described assemblage B outbreak [23] clustered together, and have high ASH, a property shared with other isolates that in majority were typed as sub-assemblage BIII by standard typing tools. In addition, the new typing scheme clustered samples from longitudinal cases together and showed them to be distinct from sporadic cases. It also revealed instances in which not all samples from a case were clustered together, which, as already discussed [7], could be explained by multi-strain infections or reinfections with a different strain over time. Interestingly, epidemiologically related cases, with a single exception, were characterized by having high ASH content. Since these longitudinal samples were from patients experiencing drug treatment failure, isolates with high ASH may have an intrinsic lower drug susceptibility than isolates with low ASH.

Subdivision of sub-assemblages BIII and BIV within assemblage B is not fully supported by analysis of classical DNA marker sequences [8,13]. We also showed that human assemblage B samples cannot be unambiguously categorized into BIII or BIV, neither using the classical marker genes (e.g., *tpi*) nor using our seven loci typing scheme. Rather, we observed a gradual differentiation from isolates with low/no ASH (homozygotes) to those with moderate ASH and finally those with high ASH (heterozygotes). Of note, this ASH pattern did not fully coincide with the subdivision of the isolates into BIII and BIV subgroups. However, it is important to note that our exploratory dendrogram (Fig 1B) showed that the two sub-assemblages BIII and BIV were equally represented by the 18 whole genome sequences derived from axenically cultured isolates. During our further marker selection process, we solely focused on obtaining enough resolution power to distinguish individual isolates. Future studies on larger collections of assemblage B genomes are urgently needed to resolve the true population structure.

Although the occurrence of ASH and its impact on standard sequence analysis have been well recognized [34], ASH positions have been largely ignored in published studies, partly because standard sequencing software tools are programmed to present a single nucleotide at any given position, rather than IUPAC codes. We originally intended to identify informative genomic regions for typing with no or low ASH content to avoid these issues. However, no such regions could be retrieved from a genome-wide search. Analysis of the selected markers showed a vast distribution of different ASH contents in the different markers, with some isolates being homozygotes (no ASH), other being heterozygotes (ASH at all marker sequences), and others being mixed (no ASH at some markers but ASH at other markers). The ASH content of each marker varies and the higher the ASH content the more markers may be heterozygote. The comparison of ASH pattern (presence/absence) between markers can already be helpful to compare or identify related samples as exemplified by the results of the samples from the Italian outbreak that showed a specific ASH pattern between markers. We conclude that it will be unlikely to identify genomic regions within the assemblage B population that show no or low ASH. As a particular genotype is defined by its allele content, it will be necessary to include this information irrespective of the procedures used to type assemblage B. Although our procedure is resource demanding (in particular the sequence analysis for ASH calling is time consuming and difficult to standardize), it is robust and ASH calling was successful with high confidence in a ring trial involving 13 partner laboratories [35].

Our study has several limitations. Genotyping of *G. duodenalis* is still not standardized and an overall formidable task. The trophozoite stage of the parasite possesses two nuclei with diploid genomes each (4N organism, alternating during replication between 4N-8N) and the excreted cyst stage contains 16N (4 nuclei with 4N) [36]. Thus, the presence of significant ASH content, as exemplified here for assemblage B reaching ASH proportions between 0–2% [5], produces ambiguous sequence datasets when using conventional Sanger sequencing approaches and to set appropriate cut off values to detect ASH with relation to the genomic polyploid state is challenging. Furthermore, *Giardia* are not routinely culturable from patient samples and genotyping approaches rely on analysis of fecal samples or purified cysts from fecal material. This may generate further biases due to potential inter- or intra-assemblage mixed infections. In this case, Sanger sequencing will only determine an “average” genotype of the sample, and it is not possible to exclude confounders like questionable reliability of typing by nested PCR in case of (low level) multi-strain infections. This could explain why samples of case 6 and case 8 did not cluster together, an issue we previously discussed after using a conventional three loci MLST approach [7]. These caveats may hinder appropriate data interpretation, in particular for epidemiological purposes. Potential alternative approaches for genotyping, e.g., fragment length analysis of markers containing simple sequence repeats, which are available for other intestinal pathogens like *Cryptosporidium parvum* [37], are not advanced for *Giardia* and warrant further investigation [38,39].

In conclusion, we provided valuable information on variable genomic regions in assemblage B genomes and developed a new typing scheme based on seven marker sequences. The sequence analysis included robust calling of ASH positions, as genotypes were represented by their allele content. As the current procedure is resource demanding, the development of more sophisticated and automated methods, such as amplicon-directed NGS approaches, will be necessary to implement more straight forward approaches for routine investigations in an epidemiological context.

## Supporting information

S1 Appendix Table A: List of samples used in the study. Table B: List of 42 identified genomic regions. Output results were ranked according to the number of Position of Interest (POI) within each of the 42 output sequences. Each POI represents the central nucleotide of a 501 bp genomic region suitable to distinguish the 18 genomic input sequences. The alignments of the 42 regions are provided in S2 Appendix. Table C: List of oligo nucleotides. (XLSX)

S2 Appendix Sequence alignments in fasta format of the 42 identified genomic regions, including Position of Interest (POI). (ZIP)

S1 Fig Scheme of typing procedure. (PDF)

S2 Fig Marker sequences of reference isolate and oligo binding sites. (PDF)

S3 Fig Alternative tree representation of dataset as shown in Fig 5 using UPGMAT calculation on distance matrix using Ridom SeqSphere software tool. (PDF)

S4 Fig Multi-spanning tree representation of dataset as shown in Fig 5 using calculations on distance matrix implemented Ridom SeqSphere software tool. Samples with a distance ≤ 55 are highlighted in grey. Samples of the same case that cluster together are circled. The three samples of case 6 and 8 that did not cluster with their related samples were not highlighted.
(PDF)

S5 Fig Copy of Fig 5, including sample names. (PDF)
