# Supplementary material for: High resolution multi-locus sequence typing scheme for Giardia duodenalis assemblage B outbreak and population analysis
Source: PLoS Negl Trop Dis. 2026 Jul 15;20(7):e0014528. doi: 10.1371/journal.pntd.0014528 (PMC13395364; doi:10.1371/journal.pntd.0014528)
Supplement: S1 Fig — (PDF) [file pntd.0014528.s003.pdf]

Input
bi-dectional Sanger sequencing from nested PCR

↓

Assemble trimmed sequences and call IUPAC code of ambigues sites

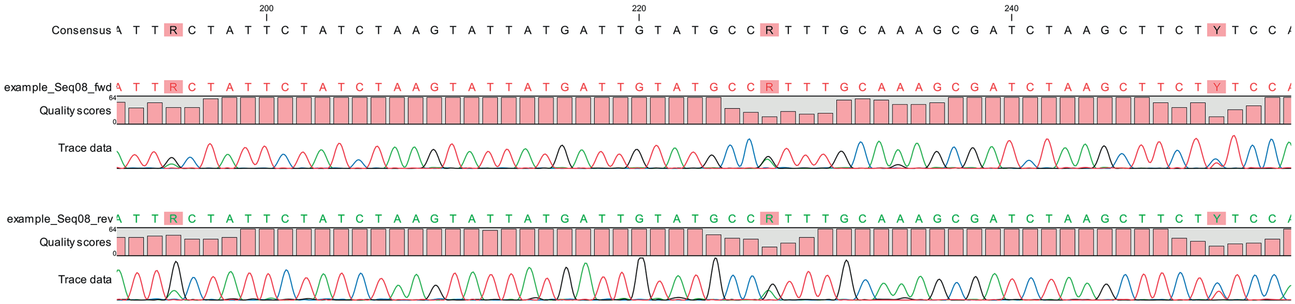

↓

Extract consensus sequence
(excluding primer sequences)

↓

Downstream analysis

↓

Multiple sequence alignment of consensus sequences
(e.g., calculate distance)

↓

Calculate pairwise distance

Isolate 1: ..TRCT..
Isolate 2: ..TGCT..
= distance 1
