# Supplementary figures and images for: High resolution multi-locus sequence typing scheme for Giardia duodenalis assemblage B outbreak and population analysis
Source: PLoS Negl Trop Dis. 2026 Jul 15;20(7):e0014528. doi: 10.1371/journal.pntd.0014528 (PMC13395364; doi:10.1371/journal.pntd.0014528)

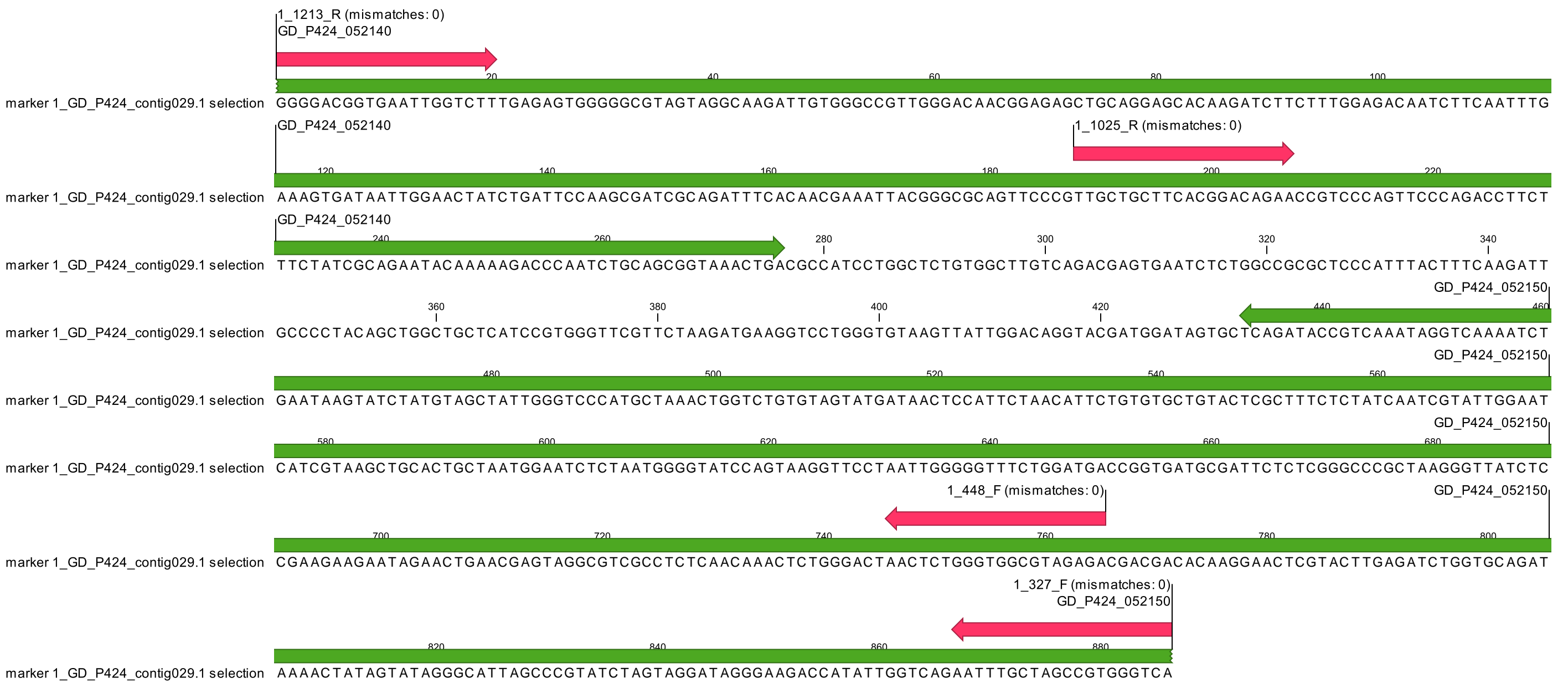

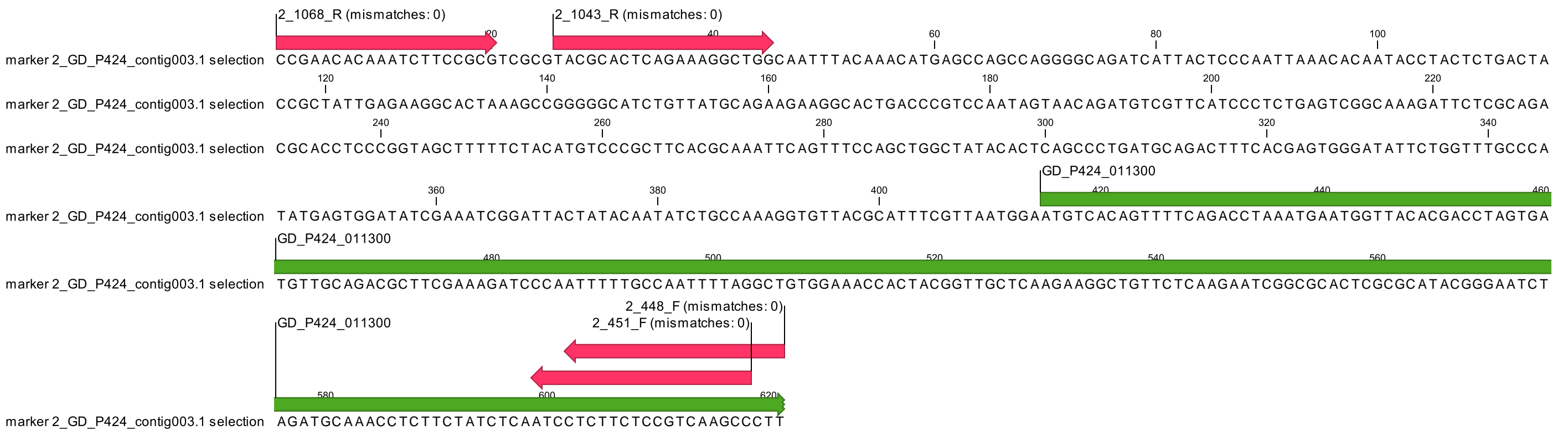

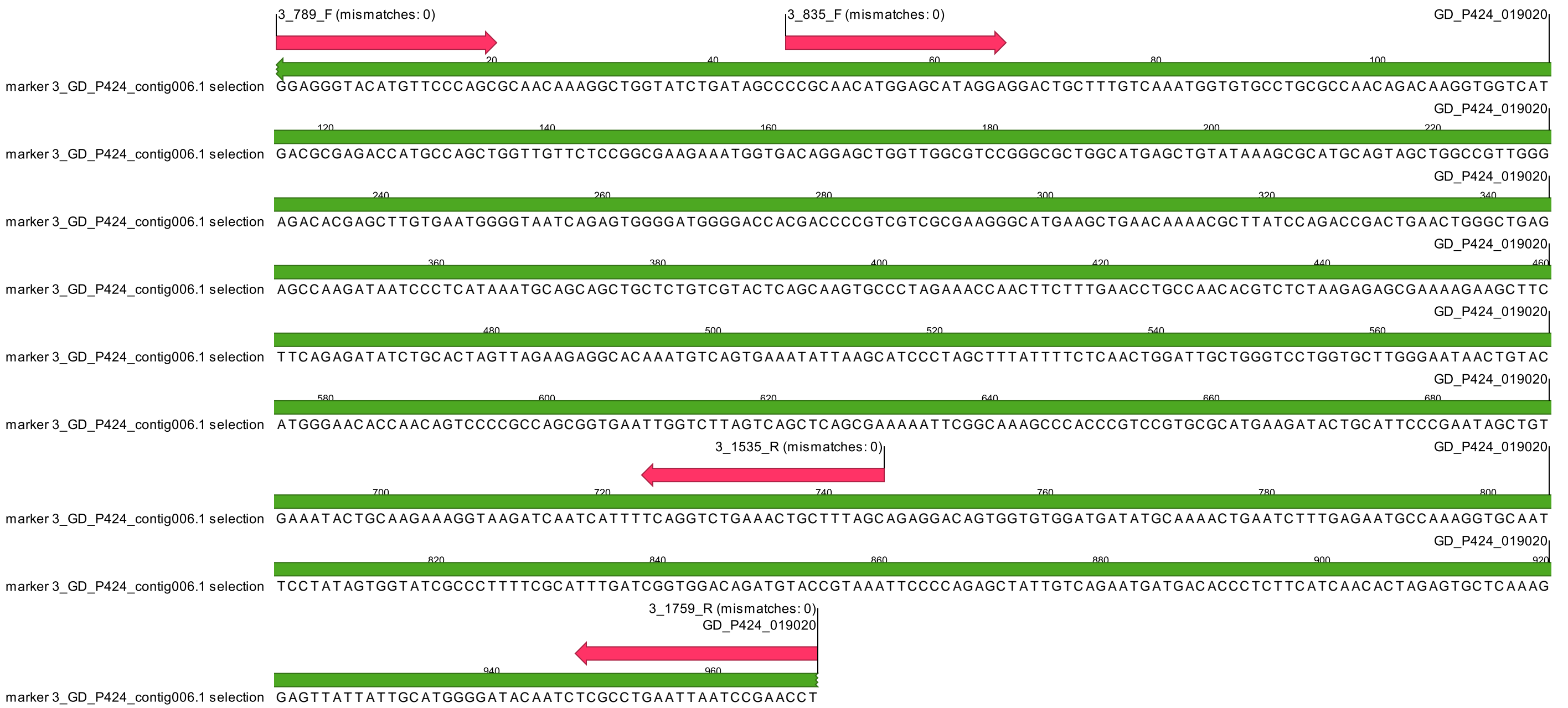

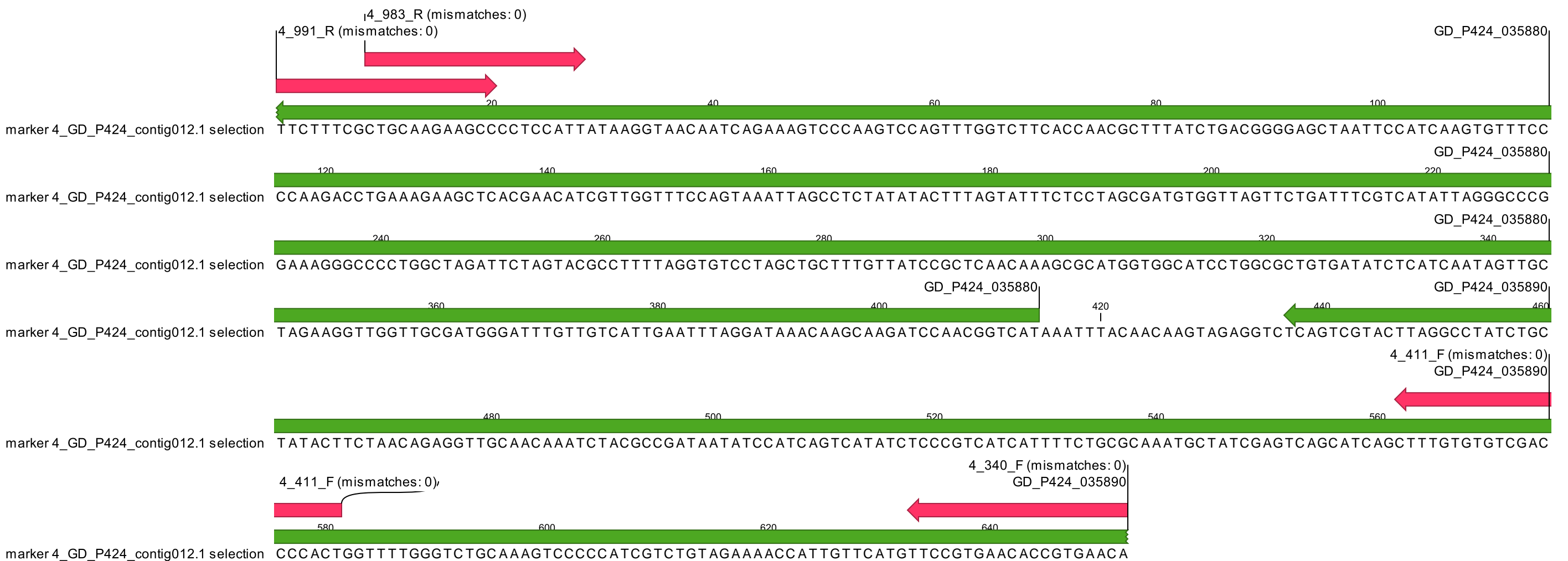

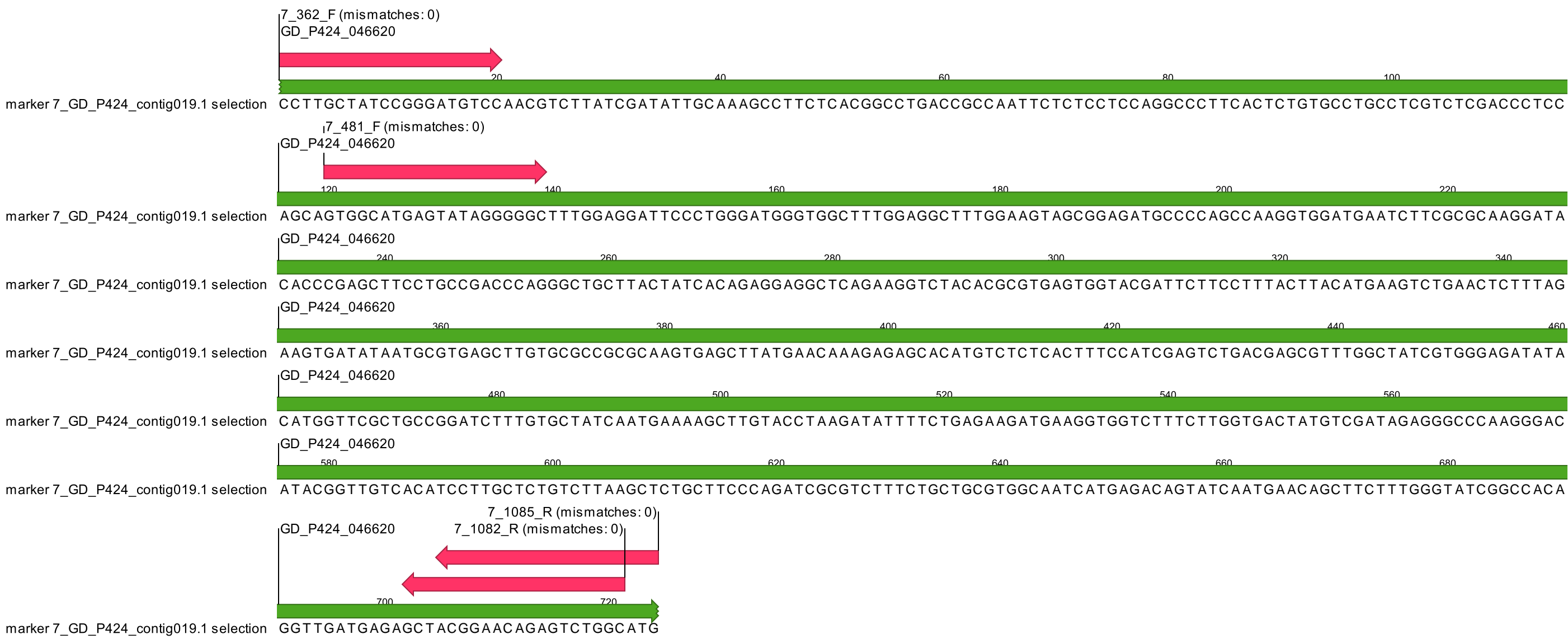

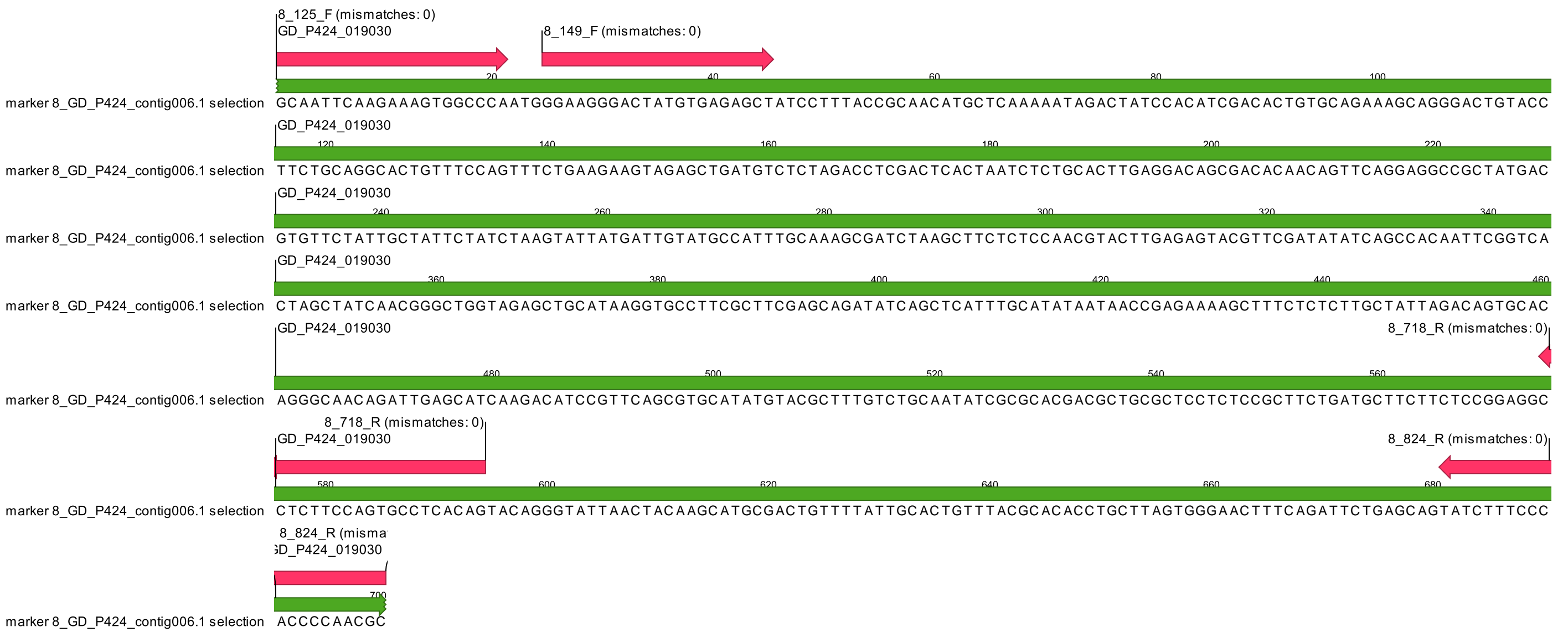

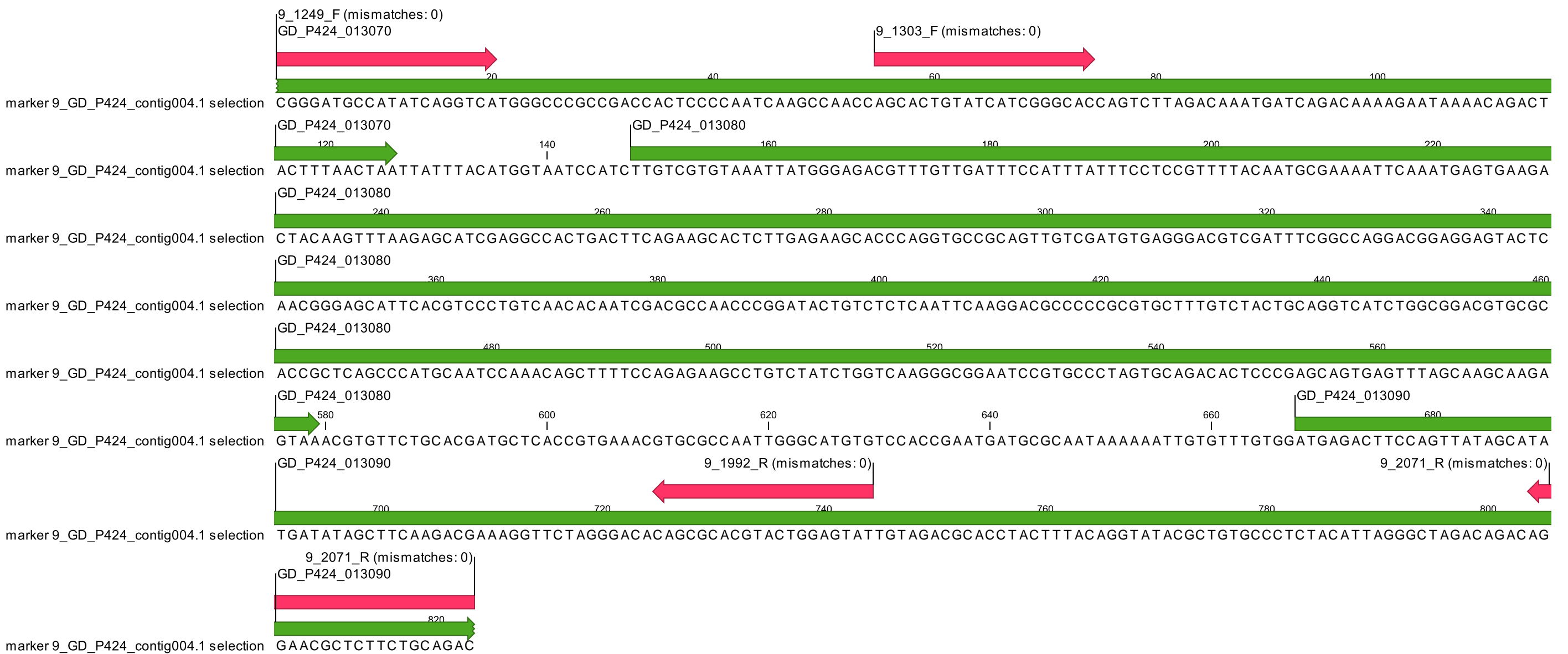

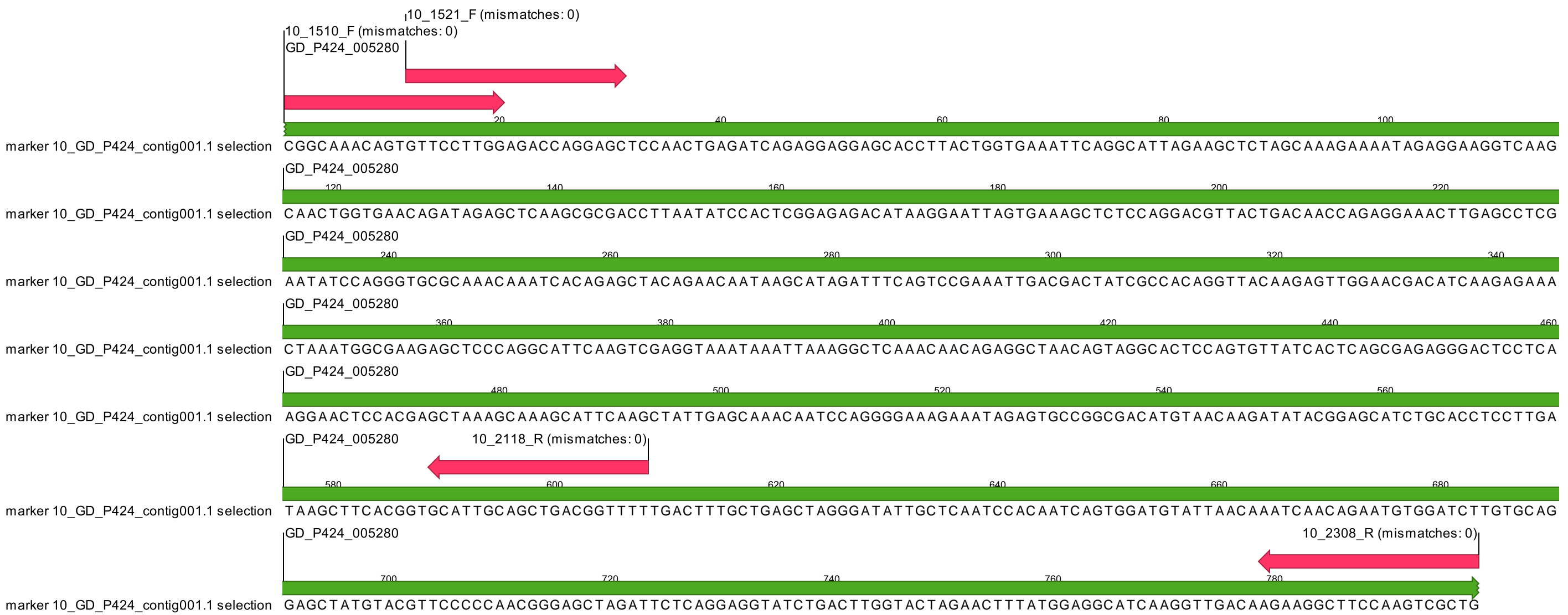

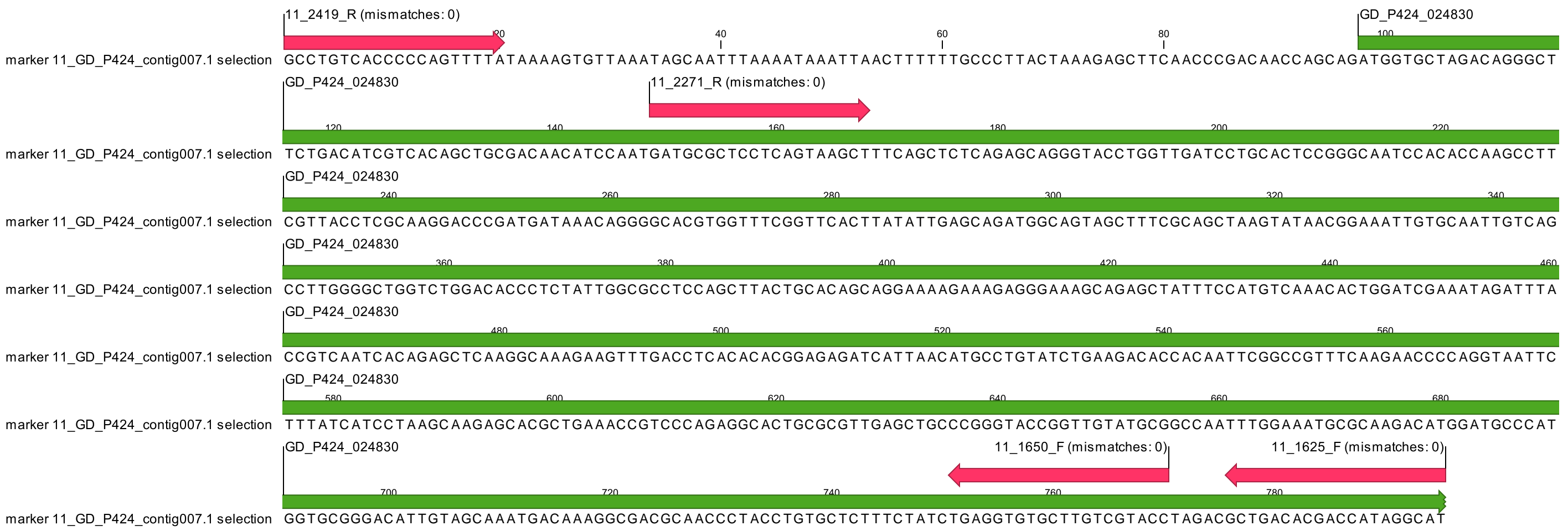

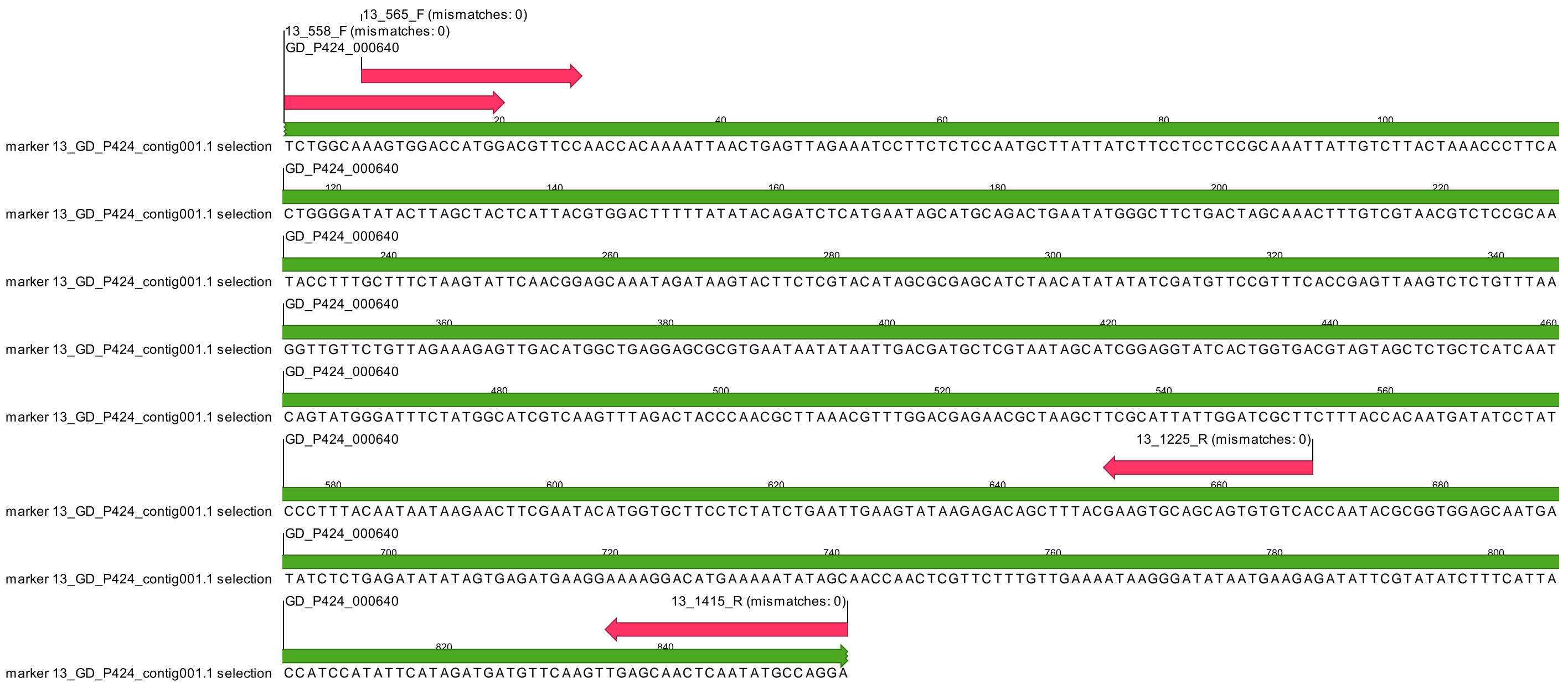

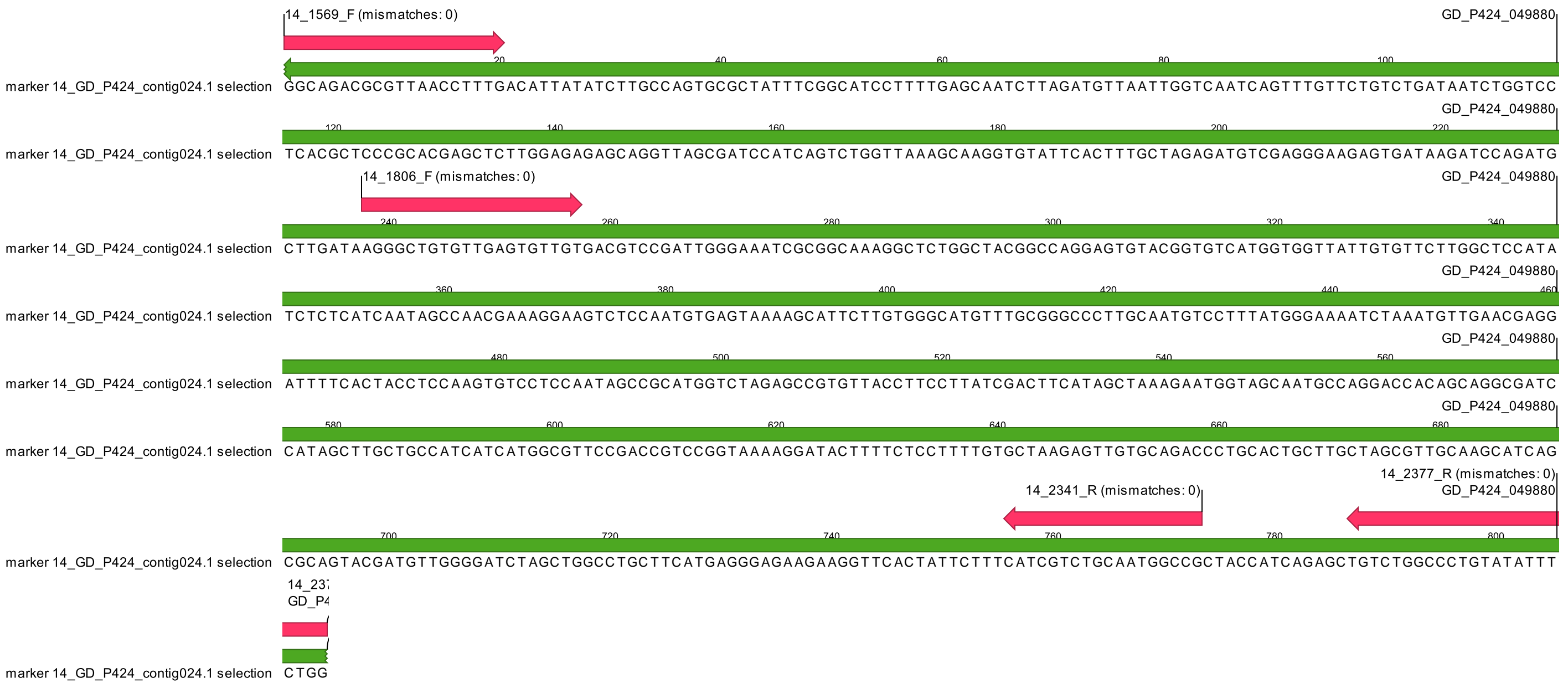

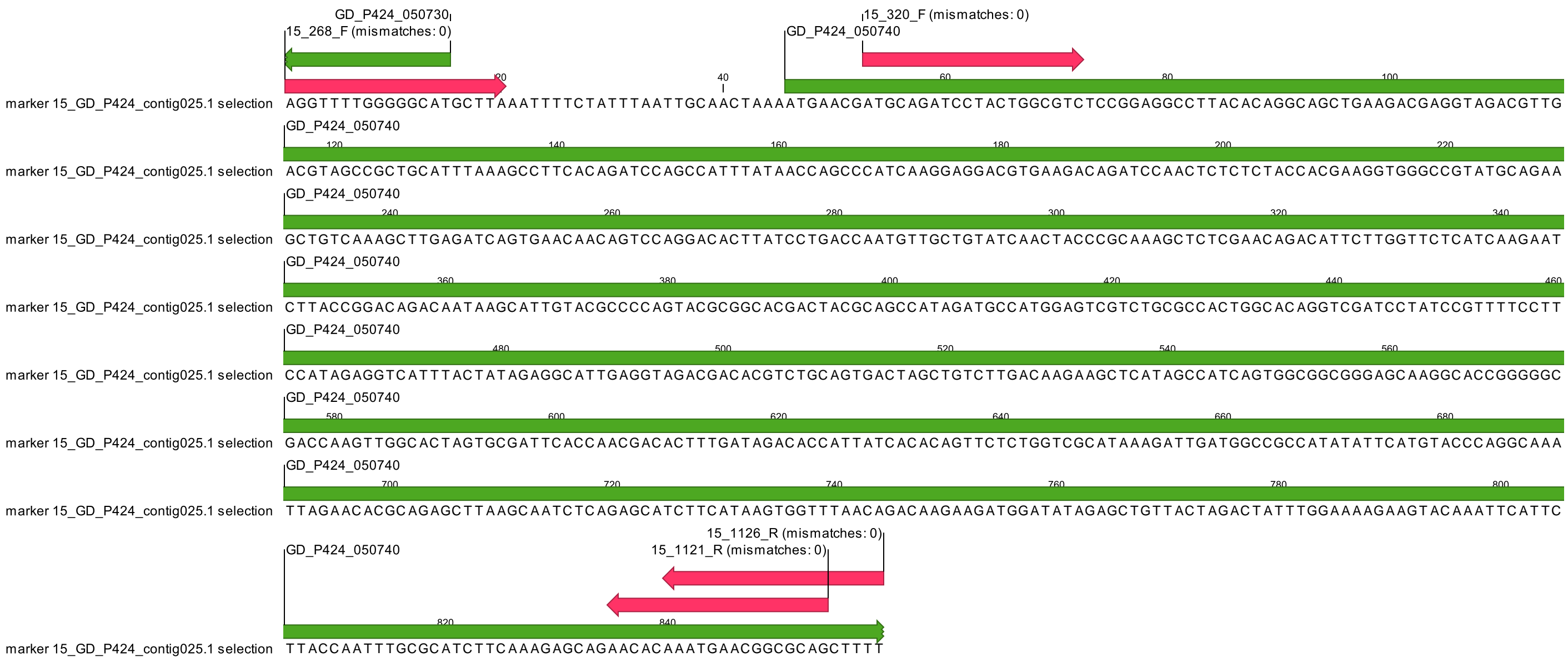

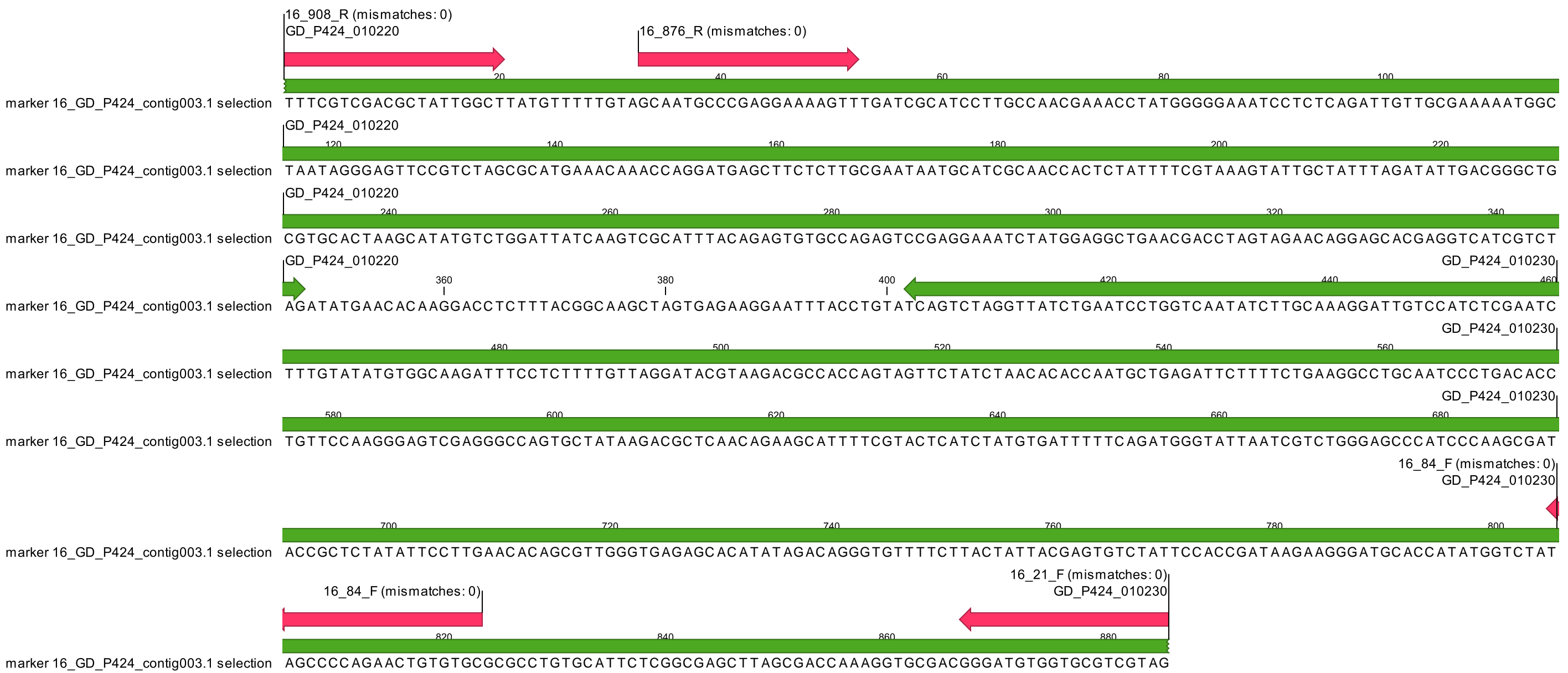

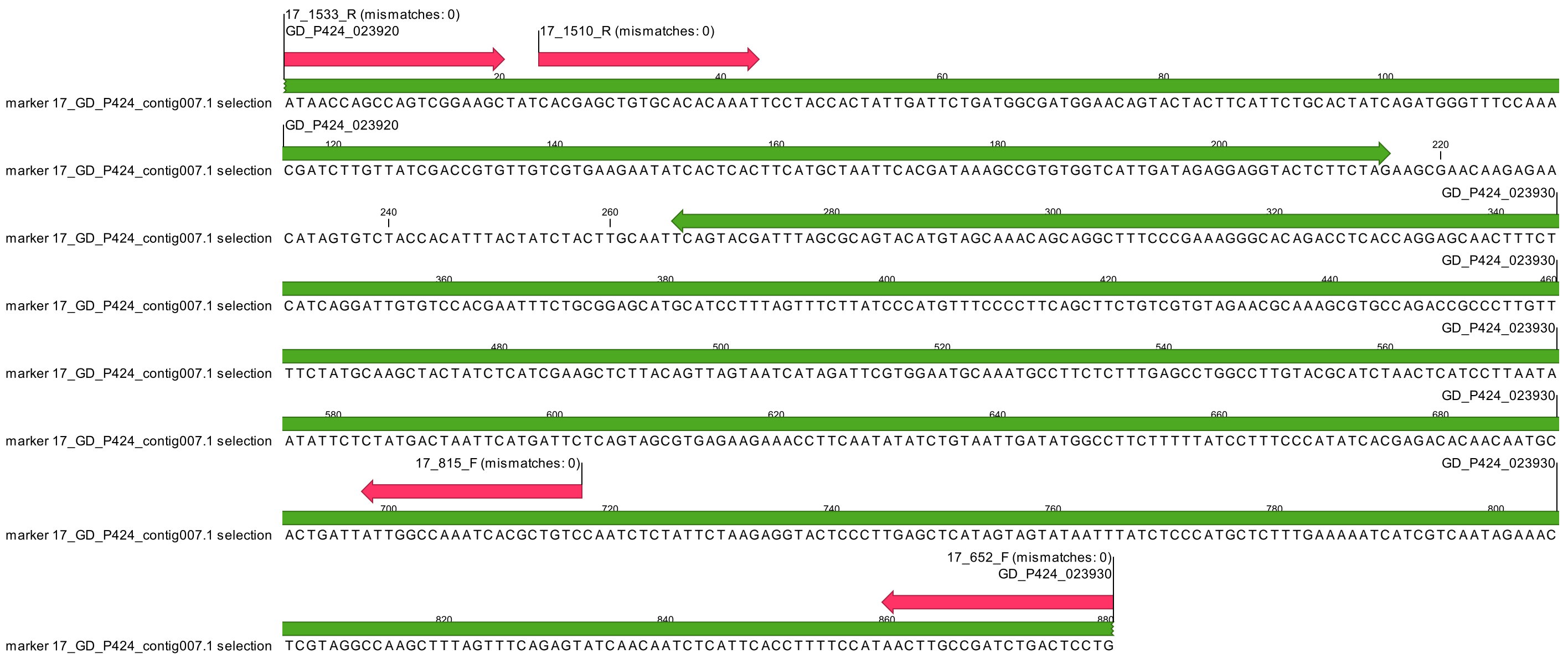

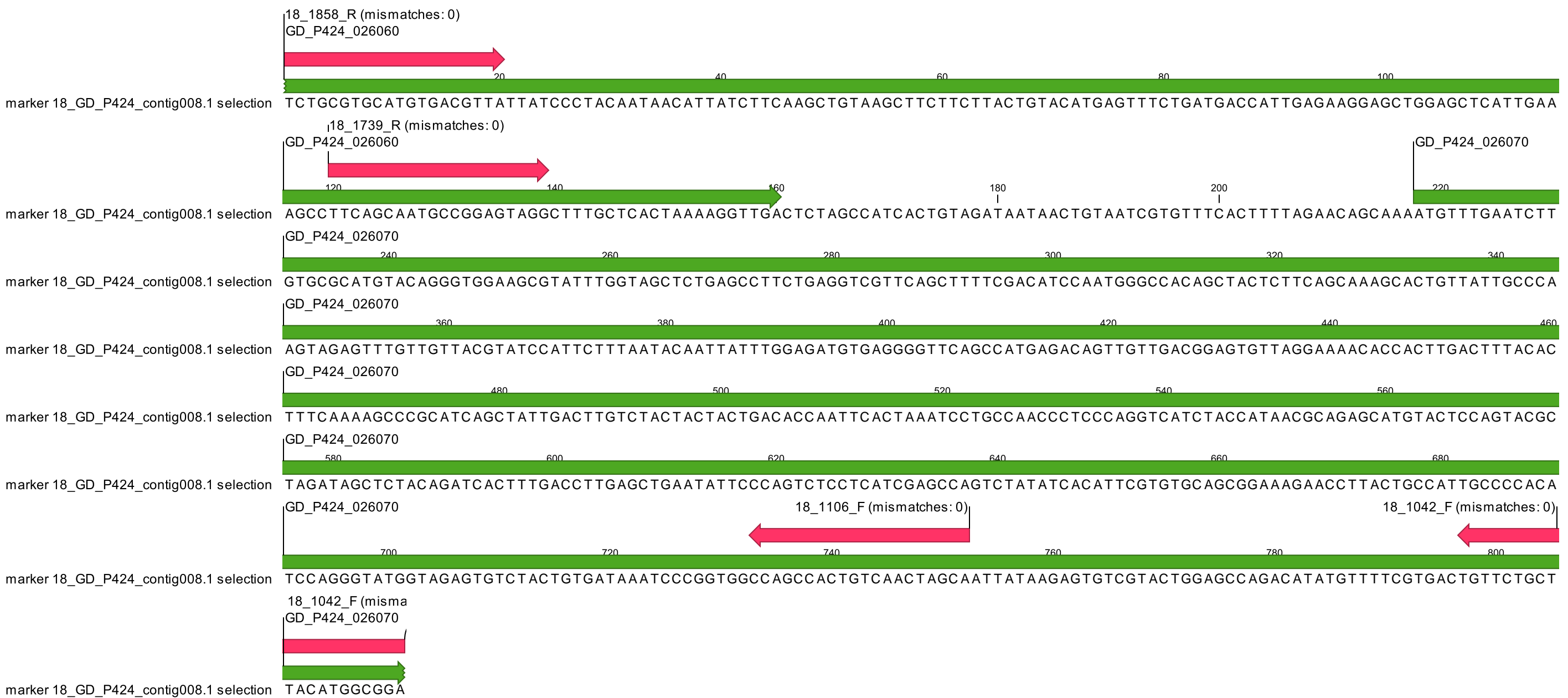

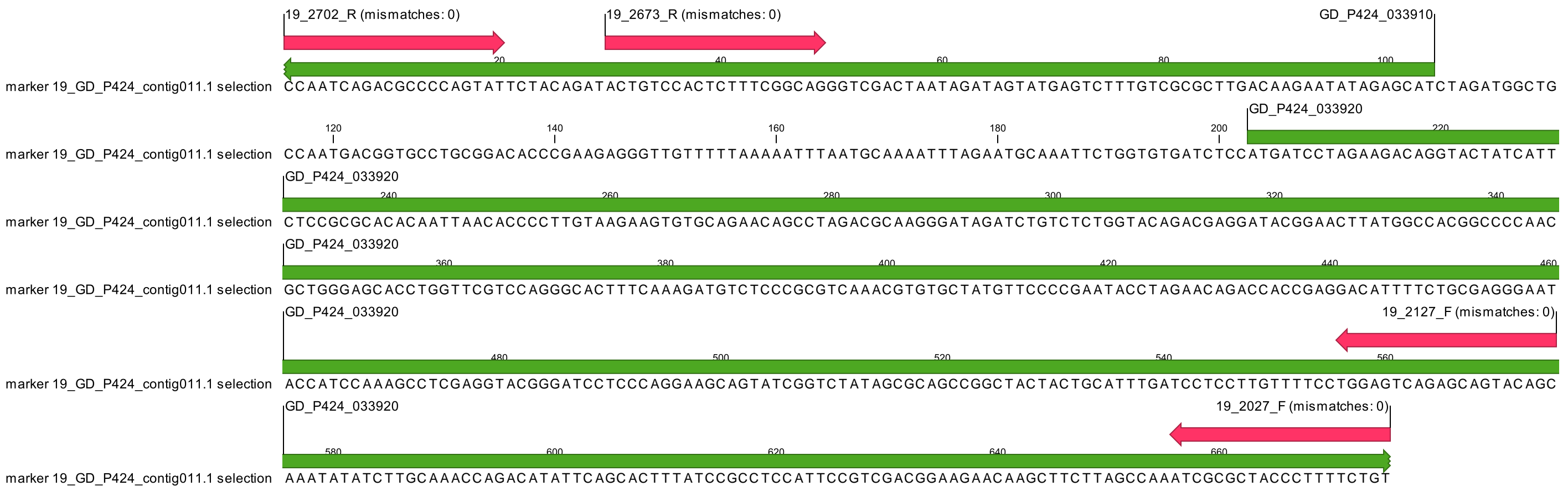

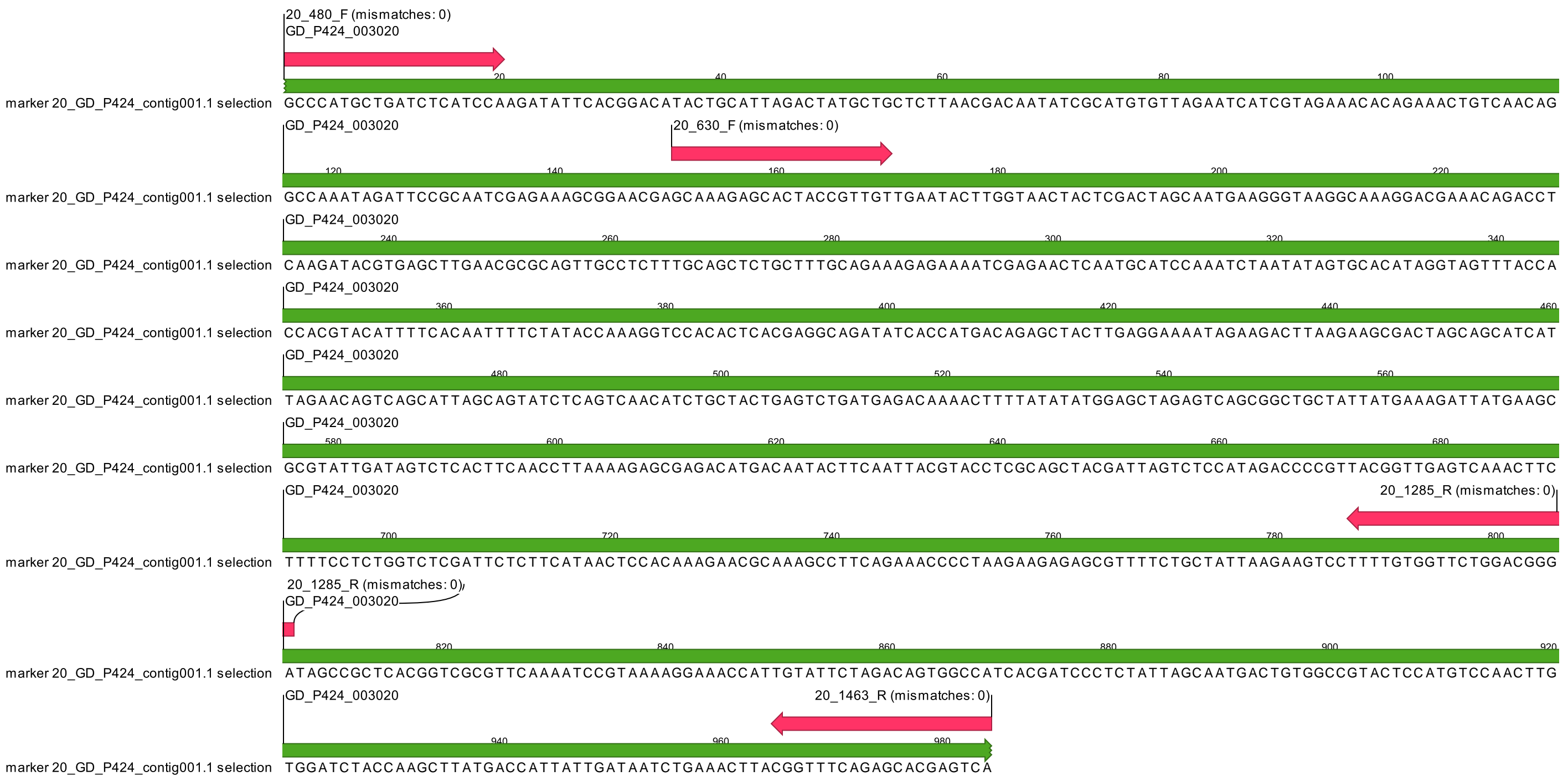

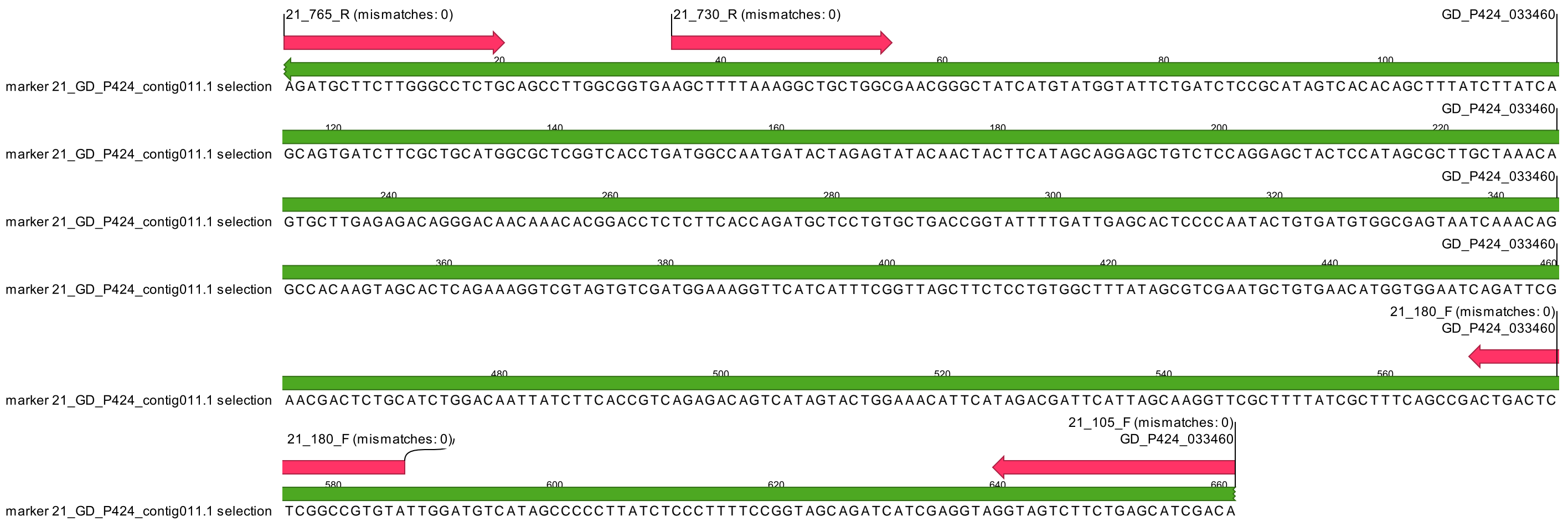

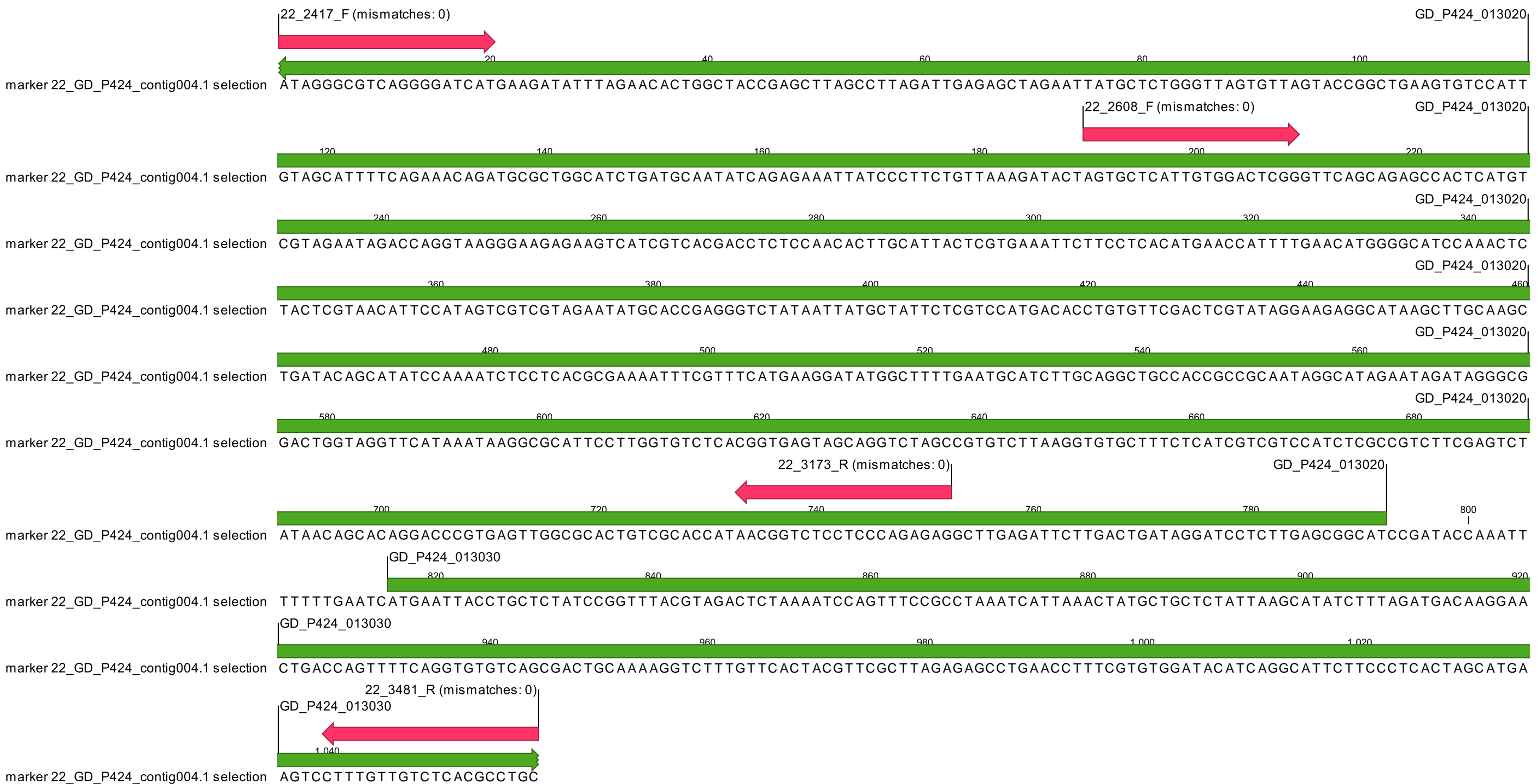

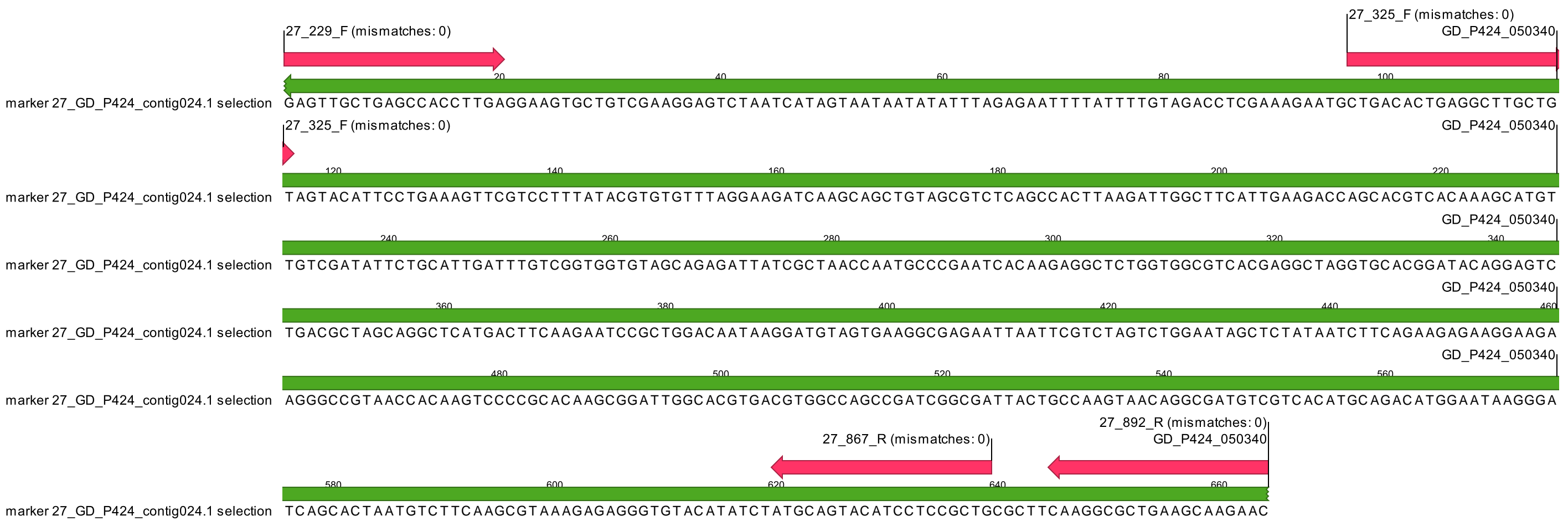

Supplement: S2 Fig — (PDF) [file pntd.0014528.s004.pdf]

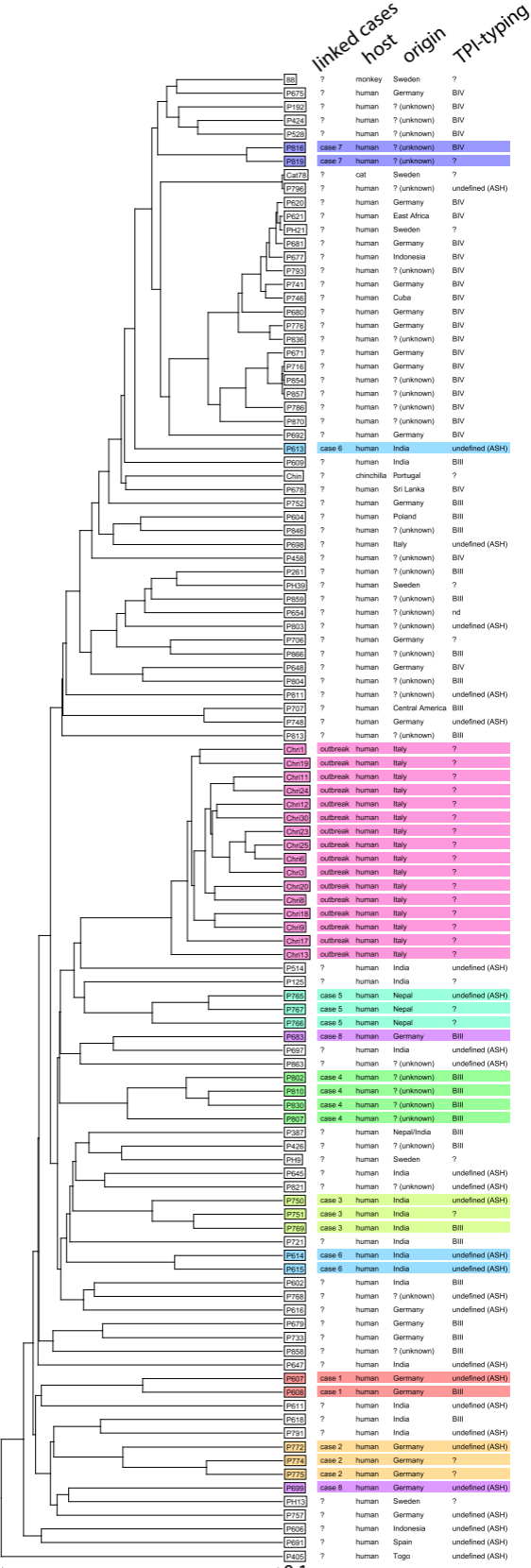

Supplement: S3 Fig — (PDF) [file pntd.0014528.s005.pdf]

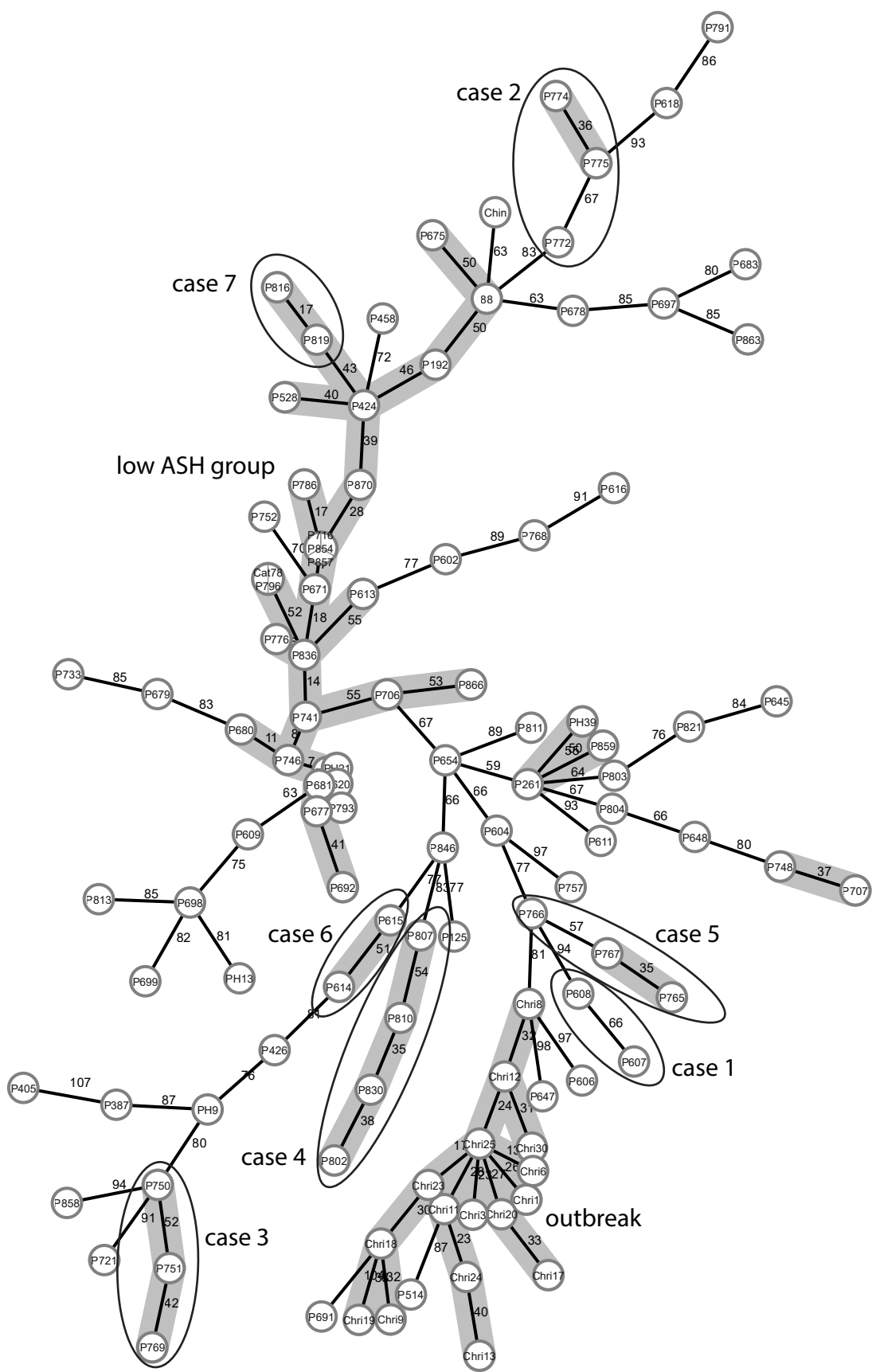

Supplement: S4 Fig — Samples with a distance ≤ 55 are highlighted in grey. Samples of the same case that cluster together are circled. The three samples of case 6 and 8 that did not cluster with their related samples were not highlighted. (PDF) [file pntd.0014528.s006.pdf]

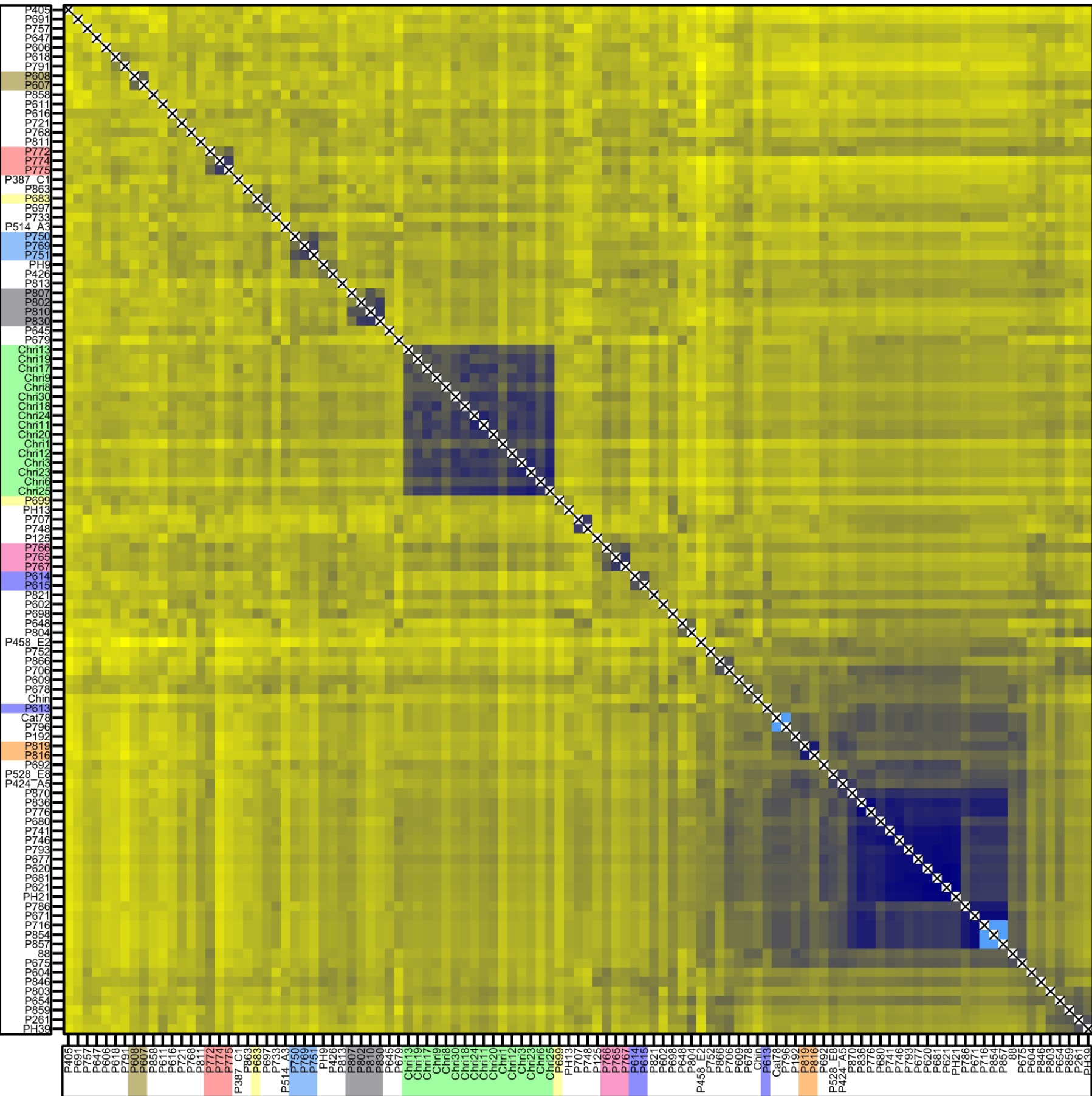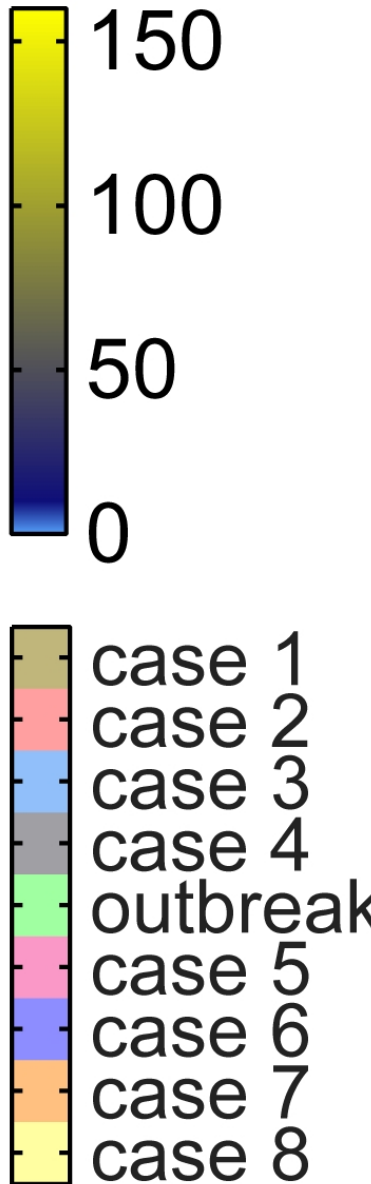

Supplement: S5 Fig — (PDF) [file pntd.0014528.s007.pdf]
